# Rejuvenated Hematopoietic Stem and Progenitor Cell-Engineered CAR-Armored Natural Killer T Cells for Malignant Pleural Mesothelioma

**DOI:** 10.34133/research.1310

**Published:** 2026-06-11

**Authors:** Yan-Ruide Li, Yichen Zhu, Zhe Li, Xinyuan Shen, Shuo Li, Yuning Chen, Zibai Lyu, Jie Huang, Nathan Y. Ma, Catherine Zhang, Annabel S. Zhao, Yanxin Tian, Xianghong Jasmine Zhou, Lili Yang

**Affiliations:** ^1^ Department of Microbiology, Immunology & Molecular Genetics, University of California, Los Angeles, Los Angeles, CA 90095, USA.; ^2^ Department of Bioengineering, University of California, Los Angeles, Los Angeles, CA 90095, USA.; ^3^ Department of Pathology and Laboratory Medicine, David Geffen School of Medicine, University of California, Los Angeles, Los Angeles, CA 90095, USA.; ^4^ Eli and Edythe Broad Centre of Regenerative Medicine and Stem Cell Research, University of California, Los Angeles, Los Angeles, CA 90095, USA.; ^5^ Jonsson Comprehensive Cancer Centre, University of California, Los Angeles, Los Angeles, CA 90095, USA.; ^6^ Molecular Biology Institute, University of California, Los Angeles, Los Angeles, CA 90095, USA.; ^7^ Parker Institute for Cancer Immunotherapy, University of California, Los Angeles, Los Angeles, CA 90095, USA.; ^8^ Goodman-Luskin Microbiome Center, University of California, Los Angeles, Los Angeles, CA 90095, USA.

## Abstract

Malignant pleural mesothelioma (MPM) is a rare and highly aggressive malignancy arising from the pleural lining, characterized by a dismal prognosis and limited therapeutic options. Mesothelin (MSLN)-directed chimeric antigen receptor (CAR)-armored T (CAR-T) cell therapies have shown encouraging preliminary outcomes; however, autologous manufacturing approaches remain constrained by logistical complexity and prolonged production timelines, which are suboptimal for patients with rapidly advancing disease. Here, we describe the development of human allogeneic interleukin-15-augmented, MSLN-specific, CAR-armored invariant natural killer T (^Allo15^MCAR-NKT) cells. These cells are generated through genetic modification of hematopoietic stem and progenitor cells, followed by a clinically guided CAR-NKT cell differentiation, maturation, and expansion process. This approach supports scalable production with high cellular yield, purity, and translational feasibility. Functionally, ^Allo15^MCAR-NKT cells exhibit robust antitumor efficacy in vitro and demonstrate robust therapeutic activity across multiple in vivo MPM xenograft models, including subcutaneous and lung metastasis models. In addition, they actively modulate the tumor microenvironment by targeting CD1d^+^ tumor-associated macrophages. Phenotypic analysis reveals a rejuvenated cellular profile, marked by low expression of exhaustion-associated and inhibitory receptors, including PD-1, TIM-3, LAG-3, CTLA-4, and TIGIT, consistent with sustained functional capacity. Importantly, these cells display a favorable safety profile, with minimal evidence of graft-versus-host disease, cytokine release syndrome, brain infiltration or neurotoxicity, and no detectable off-tumor effects. Collectively, these findings support the development of a clinically translatable, off-the-shelf CAR-NKT cell therapy for the treatment of MPM.

## Introduction

Malignant pleural mesothelioma (MPM) is a rare and aggressive pleural malignancy driven primarily by chronic asbestos exposure, which induces persistent inflammation and genomic damage that culminate in mesothelial tumorigenesis [1]. Although asbestos use is now regulated, MPM incidence remains substantial, with approximately 3,000 new cases diagnosed annually and a latency period of 30 to 50 years [1,2]. Despite decades of clinical research, therapeutic progress remains limited, with standard treatments, such as surgery, chemotherapy, and radiation, offering only modest benefit and a median life expectancy of 12 to 21 months [3]. These statistics underscore the critical need for the development of novel, effective, and readily accessible immunotherapeutic strategies.

Chimeric antigen receptor (CAR)-armored T (CAR-T) cell therapy has transformed the therapeutic landscape of hematologic malignancies and is increasingly being investigated for application in solid tumors, including MPM [4–7]. Mesothelin (MSLN), a cell surface glycoprotein, is expressed in the majority of MPM cases (approximately 80% to 90%), although expression levels can vary considerably across tumors and histological subtypes [8,9]. Its relatively restricted distribution in normal tissues and prevalent expression in mesothelioma make it an attractive target for CAR-T cell therapy. In addition, MSLN has been functionally implicated in tumor progression, facilitating tumor cell adhesion, migration, and metastasis through interactions with CA125/MUC16 and activation of downstream signaling pathways such as phosphatidylinositol 3-kinase (PI3K)/AKT and mitogen-activated protein kinase (MAPK), thereby contributing to the aggressive biology of mesothelioma [10,11]. These functional roles contribute to the aggressive nature of MPM and further underscore the therapeutic relevance of targeting MSLN. Initial clinical evaluations of MSLN-directed CAR-T (MCAR-T) cells have reported encouraging therapeutic activity [12,13], with regional administration strategies, such as intrapleural delivery, being employed to improve tumor localization and reduce systemic toxicity [14,15]. However, current approaches are limited by the logistical and clinical drawbacks of autologous manufacturing, including high cost, long production time, and patient-specific variability. These challenges pose barriers to timely access, especially in patients with rapidly progressive disease, such as MPM [16–18].

Human invariant natural killer T (NKT) cells are a rare subset of innate-like T lymphocytes that integrate features of both innate and adaptive immune responses and display potent antitumor effector functions [19–23]. Relative to conventional αβ T cells, NKT cells exhibit distinct properties that render them particularly suitable for CAR engineering for cancer therapy. These include efficient trafficking to solid tumors mediated by high chemokine receptor expression, the capability to remodel the solid tumor microenvironment (TME) through targeting of suppressive myeloid populations, and the ability to eradicate malignant cells via CAR-dependent, invariant T cell receptor (TCR)-mediated, and natural killer receptor (NKR)-driven multimodal cytotoxic mechanisms [22,24–29]. Critically, their restricted recognition of the nonpolymorphic CD1d molecule enables their safe use in allogeneic settings without inducing graft-versus-host disease (GvHD) [30–33]. However, their clinical application has been limited by their low frequency in human peripheral blood (approximately 0.001% to 1%) and the risk of contamination with conventional αβ T cells during ex vivo engineering and expansion from donor peripheral blood mononuclear cells (PBMCs) [34,35].

To overcome these limitations, we established a clinically guided, feeder-free biotechnology platform for the generation of therapeutic-scale allogeneic CAR-NKT cells derived from cord blood (CB) CD34^+^ hematopoietic stem and progenitor cells (HSPCs) [28]. Building on this, we now report the production of an optimized allogeneic, interleukin-15 (IL-15)-augmented, MSLN-directed CAR-NKT (^Allo15^MCAR-NKT) cell product with the goal of achieving safe and effective treatment of MPM. Here, we perform a comprehensive preclinical evaluation of ^Allo15^MCAR-NKT cells, including molecular profiling, in vitro and in vivo antitumor efficacy, mechanism of action, persistence, biodistribution, and safety. We demonstrate that ^Allo15^MCAR-NKT cells exhibit potent MPM tumor killing capacity through multimodal tumor-targeting mechanisms, display low levels of exhaustion with a rejuvenated functional potential, remodel the MPM TME, and maintain a favorable safety profile. Our results support ^Allo15^MCAR-NKT as a scalable and clinically translatable allogeneic cell therapy for addressing the unmet medical need in MPM.

## Results

### Profiling the MPM tumor antigen landscape reveals the therapeutic potential of CAR-NKT cells

**Fig. 1.**
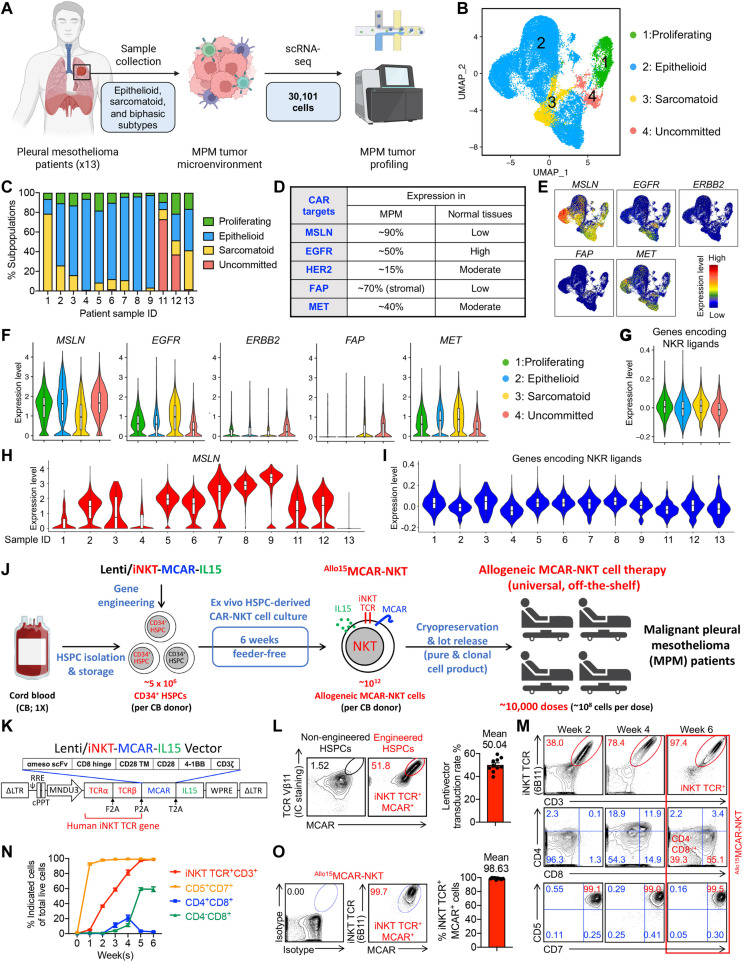
Profiling the MPM tumor antigens and generating allogeneic IL-15-enhanced MCAR-engineered NKT (^Allo15^MCAR-NKT) cells for targeted therapy. (A to I) Profiling the MPM tumor antigens using scRNA-seq analysis. (A) Experimental design to profile MPM tumor cells using scRNA-seq. A total of 13 primary MPM patient samples were included. (B) Combined UMAP plot showing the formation of 4 major cell clusters. (C) Bar graphs showing the cell cluster proportions of the individual patient samples. Patient sample 10 was excluded from the analysis due to the absence of malignant cells. (D) Table showing the potential CAR targets for MPM and their expression in MPM tumors and normal tissues. (E) UMAP plots showing the gene expression distribution of the indicated CAR targets. (F and G) Violin plots showing the expression distribution of genes encoding the indicated CAR targets (F) and NKR ligands (G) compared among the 4 cell clusters. (H and I) Violin plots showing the expression distribution of genes encoding the indicated CAR targets (H) and NKR ligands (I) in the individual patient samples. (J to O) Generation of ^Allo15^MCAR-NKT cells. (J) Schematics showing the generation of ^Allo15^MCAR-NKT cells. HSPC, hematopoietic stem and progenitor cell; MCAR, mesothelin-targeting CAR. (K) Schematics showing the design of indicated lentivector. Δ LTR, self-inactivating long-term repeats; MNDU3, internal promoter derived from the MND retroviral LTR U3 region; φ , packaging signal with the splicing donor and splicing acceptor sites; RRE, rev-responsive element; cPPT, central polypurine tract; WPRE, woodchuck responsive element; αmeso, anti-mesothelin; scFv, single-chain variable fragment; TM, transmembrane. (L) Fluorescence-activated cell sorting (FACS) analyses of the lentivector transduction rate on CD34^+^ HSPCs (*n* = 10; *n* indicates different CB donors). The intracellular iNKT TCR was stained using a TCR Vβ11 antibody, and MCAR was stained using an anti-mouse IgG F(ab′)2 antibody. (M) FACS monitoring of the generation of ^Allo15^MCAR-NKT cells. The surface iNKT TCR was stained using a 6B11 monoclonal antibody. (N) Percentage of the indicated cells in total live cells over time (*n* = 4; *n* indicates different CB donors). (O) FACS analyses of the purity of ^Allo15^MCAR-NKT cells (*n* = 10; *n* indicates different CB donors). Representative of 1 (A to I) and >10 (J to O) experiments. Data are presented as the mean ± standard error of the mean (SEM).

We first analyzed primary MPM patient-derived tumor cells to define the antigen landscape and guide the design of targeted CAR-engineered cell therapies. Single-cell RNA sequencing (scRNA-seq) data from 13 treatment-naïve MPM patients representing all 3 histologic subtypes (i.e., epithelioid, sarcomatoid, and biphasic subtypes) were integrated to capture tumor heterogeneity and diversity (Fig. 1A) [36]. Our analysis focused on tumor antigen profiling, particularly antigens relevant for CAR-based therapies.

MPM tumor cells segregated into 4 major transcriptional clusters: proliferating, epithelioid, sarcomatoid, and uncommitted populations (Fig. 1B) [36,37]. The relative proportions of these clusters varied substantially across patients, highlighting the marked interpatient and intratumoral heterogeneity characteristic of MPM (Fig. 1C). We examined the expression of 5 leading CAR targets currently under clinical or preclinical investigation for MPM, including MSLN, epidermal growth factor receptor (EGFR), human epidermal growth factor receptor 2 (HER2), fibroblast activation protein (FAP), and mesenchymal–epithelial transition factor (MET) (Fig. 1D) [38,39]. Among these, MSLN emerged as the most favorable target, given its high and consistent expression on MPM tumor cells and relatively low expression in normal tissues, supporting a favorable therapeutic window (Fig. 1D). In contrast, FAP was predominantly expressed by stromal cells rather than tumor cells, consistent with its role as a microenvironment target (Fig. 1D) [40].

Both uniform manifold approximation and projection (UMAP) and violin plot analyses confirmed that MSLN expression was markedly higher than that of other CAR targets across tumor cells (Fig. 1E and F). Notably, MSLN expression was enriched in epithelioid and sarcomatoid tumor populations compared with proliferating cells, suggesting subtype-dependent antigen density (Fig. 1E and F). Consistent with these findings, prior clinical studies have shown that MSLN overexpression promotes mesothelioma cell invasion and disease progression, further supporting MSLN as a rational therapeutic target [38,39]. Together, these data indicate that MSLN represents the most robust antigen for CAR-based therapy in MPM.

In addition to CAR targets, we observed that MPM tumor cells expressed multiple ligands for NKRs, with comparable expression across the 4 tumor subtypes (Fig. 1G). These findings suggest that MPM cells may also be susceptible to NKR-mediated cytotoxicity through receptors such as NKG2D and DNAM-1. Importantly, analysis at the individual patient level revealed variable but concurrent expression of MSLN and NKR ligands, indicating that dual targeting strategies could be particularly effective (Fig. 1H and I).

CAR-NKT cells offer a unique therapeutic approach by combining CAR-dependent recognition with intrinsic NKR-mediated cytotoxicity, enabling simultaneous targeting of multiple tumor-associated antigens [34,41]. Based on this antigen landscape profiling and previously established allogeneic CAR-NKT cell platform [28], we developed MSLN-targeted CAR-NKT cells for MPM therapy (Fig. 1J). Moreover, the robustness and flexibility of our platform allow for straightforward adaptation to alternative targets, including EGFR, HER2, FAP, and MET, supporting the broader applicability of this strategy.

### ^Allo15^MCAR-NKT cells can be efficiently generated using an ex vivo feeder-free differentiation system, yielding a highly pure and scalable cell product

Cryopreserved, commercially sourced CB-derived CD34^+^ HSPCs were obtained from vendors such as HemaCare. These HSPCs underwent a 6-week, feeder-free ex vivo culture to generate an off-the-shelf ^Allo15^MCAR-NKT cell product intended to treat patients with MPM (Fig. 1J). This HSPC-based ex vivo differentiation platform has been previously established for the generation of allogeneic CAR-NKT cells targeting hematologic malignancies, including CD19^+^ leukemia and lymphoma, BCMA^+^ multiple myeloma (MM), and CD33^+^ and CD70^+^ acute myeloid leukemia and myelodysplastic syndromes (Fig. S1A) [28,42,43]. In the present study, we adapt this approach to target solid tumors, with a particular emphasis on MSLN^+^ MPM.

To efficiently produce ^Allo15^MCAR-NKT cells, human HSPCs were transduced with a single lentiviral construct, Lenti/iNKT-MCAR-IL-15 (Fig. 1J). The vector construct was designed to encode 3 essential functional components. First, it included the invariant NKT TCR α and β chains, derived from healthy donor PBMC-isolated NKT cells, which have been previously validated to support both autologous and allogeneic human NKT cell generation and functional activity [44,45]. Second, the construct incorporated an MSLN-directed third-generation CAR comprising the clinically validated SS1 single-chain variable fragment (scFv) for antigen recognition, linked to intracellular signaling domains from CD28 and 4-1BB, thereby enabling enhanced T cell activation, costimulation, and persistence [46]. Third, the vector encoded human soluble IL-15, a cytokine known to promote NKT cell survival, proliferation, and metabolic fitness, as well as to enhance in vivo expansion and antitumor efficacy of both PBMC-derived and HSPC-derived CAR-NKT cells (Fig. 1K) [28,42,47,48]. The lentivector transduction was highly efficient, with >50% of CD34^+^ HSPCs expressing the integrated construct (Fig. 1L). Importantly, because all 3 functional components were encoded within the same lentiviral vector, each transduced HSPC expressed the full set of transgenes, eliminating the need for further cell purification or selection.

After 48 h of culture in HSPC culture medium, transduced CD34^+^ HSPCs were subjected to a feeder-free, 5-stage ex vivo differentiation protocol spanning 6 weeks (Fig. 1J) [28,42]. During this differentiation process, developing NKT cells progressed through defined thymocyte-like developmental stages, beginning with a CD4^−^CD8^−^ double-negative (DN) phase, followed by transition to a CD4^+^CD8^+^ double-positive (DP) stage characterized by TCR rearrangement and selection (Fig. 1M and N). Subsequently, the cells underwent lineage commitment and maturation into either CD8^+^ single-positive (SP) or DN T cell subsets, consistent with invariant NKT cell developmental pathways (Fig. 1M and N). This maturation process was accompanied by the acquisition of functional markers, including invariant TCR expression and NKRs, indicative of fully differentiated and functionally competent NKT cells [49]. The generated CD8^+^ SP subset is particularly relevant for cancer immunotherapy due to its enhanced pro-inflammatory profile and superior cytotoxic potential [19,50–52].

By the end of the differentiation protocol, the resulting ^Allo15^MCAR-NKT cells achieved a high purity (>97%), as evidenced by robust coexpression of the invariant TCR and MSLN-directed CAR (Fig. 1O). Additionally, the culture process supported substantial cell expansion, with approximately 300-fold expansion in stage 1, 10-fold in stage 2, 15-fold in stage 3, and 150-fold in stage 4 (Fig. S1A to C). Starting from a single CB donor unit, typically containing approximately 5 × 10^6^ CD34^+^ HSPCs, this manufacturing process enables expansion to greater than 10^12 Allo15^MCAR-NKT cells, sufficient to support the production of an estimated 1,000 to 10,000 clinical doses (Fig. 1J and Fig. S1B and C) [53–55].

Three strategies were evaluated for stage 4 expansion: (a) αCD3/αCD28 antibody stimulation, (b) α-galactosylceramide (αGC)-loaded PBMCs, and (c) artificial antigen-presenting cells (aAPCs) (Fig. S1A, D, and E). Among these, the αCD3/αCD28 antibody-based method is fully feeder-free, offering a streamlined and clinically favorable approach for translational development [56]. Although the αGC/PBMC and aAPC-based strategies are feeder-dependent, they remain viable for clinical application, as evidenced by multiple clinical trials that have employed these materials safely [47,48,57–59]. The expansion efficiency was comparable between the 3 expansion methods, with no statistically significant differences in fold expansion observed (Fig. S1D). All 3 strategies yielded high transduction purity, with over 98% of cells coexpressing the invariant NKT TCR and the MCAR, confirming the robustness and reproducibility of the differentiation process (Fig. S1E). Furthermore, the serum-free compatibility of the aAPC-based method was validated by comparing cultures with and without serum supplementation (Fig. S1A). No significant differences in expansion were observed, demonstrating the protocol’s effectiveness in a serum-free environment (Fig. S1F).

Enzyme-linked immunosorbent assay (ELISA) analyses confirmed that ^Allo15^MCAR-NKT cells produce IL-15 (Fig. S1G and H); however, the level of endogenous IL-15 is insufficient to support their normal in vitro expansion during the final culture stage (Fig. S1I and J). Consequently, supplementation with cytokines, specifically IL-7 and IL-15, is required to achieve robust growth (Fig. S1A). These findings also demonstrate that ^Allo15^MCAR-NKT cells do not exhibit dysregulated or autonomous proliferation in the absence of exogenous cytokine support, further supporting their safety profile.

Collectively, these findings highlight that the integration of HSPC genetic engineering with a clinically guided, feeder-free ex vivo differentiation process facilitates the reproducible production of ^Allo15^MCAR-NKT cells, characterized by high yield, purity, and manufacturing consistency. The resulting cell product demonstrates reproducible expansion across different manufacturing strategies, and compatibility with serum-free, feeder-free conditions, underscoring its strong potential for clinical translation as an off-the-shelf immunotherapy for solid tumors.

### ^Allo15^MCAR-NKT cells exhibit a hybrid T and NK cell phenotype, marked with strong effector signatures, robust proliferative capacity, and potent cytotoxic programs

**Fig. 2.**
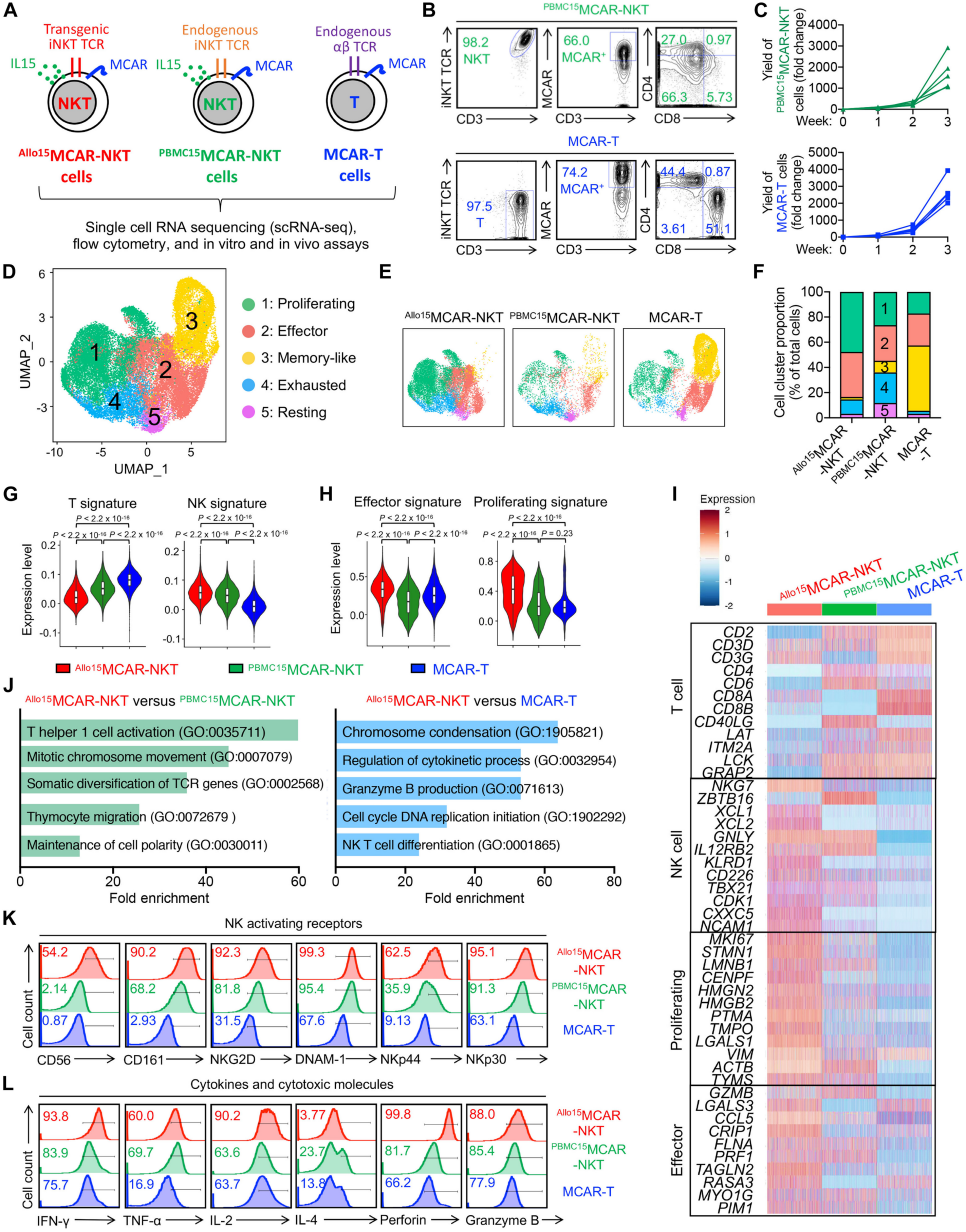
^Allo15^MCAR-NKT cells exhibit a hybrid T/NK cell phenotype, marked with strong effector signatures, robust proliferative capacity, and potent cytotoxic programs. (A) Schematic illustration comparing ^Allo15^MCAR-NKT cells with 2 benchmark controls: (1) healthy donor PBMC-derived IL-15-enhanced MCAR-NKT (^PBMC15^MCAR-NKT) cells, and (2) PBMC-derived conventional MCAR-T cells. (B) FACS plots showing the generation and characterization of ^PBMC15^MCAR-NKT and MCAR-T cells. (C) Yield of ^PBMC15^MCAR-NKT and MCAR-T cells (*n* = 4; *n* indicates different PBMC donors). (D to J) Gene profiling of the 3 therapeutic cells using scRNA-seq. (D) Combined UMAP plot showing the formation of 5 major cell clusters: Proliferating, Effector, Memory-like, Exhausted, and Resting. Total cells combined from all therapeutic cell samples are included. Each dot represents a single cell and is colored according to its cell cluster assignment. (E) Individual UMAP plots showing cell cluster composition of the indicated therapeutic cell samples. (F) Bar graph showing the cell cluster proportions of the indicated therapeutic cell samples. (G and H) Violin plots showing the expression distribution of T and NK cell gene signatures (G), as well as effector and proliferating gene signatures (H) in the indicated individual samples. (I) Heatmap showing the expression of representative signature genes. Each column indicates an individual cell. Each row indicates an individual gene. (J) Pathway analyses of differentially expressed genes comparing ^Allo15^MCAR-NKT cells with ^PBMC15^MCAR-NKT cells (left) or MCAR-T cells (right). (K and L) FACS detection of NK receptor (NKR) expression (K), as well as intracellular cytokine and cytotoxic molecule production (L) of ^Allo15^MCAR-NKT, ^PBMC15^MCAR-NKT, and MCAR-T cells. Representative of 1 (D to J) and over 5 (A to C, K, and L) experiments. *P* values of violin plots were determined by Wilcoxon rank sum test (G and H).

We first analyzed the phenotype and functionality of ^Allo15^MCAR-NKT cells in parallel with 2 benchmark controls: (a) healthy donor PBMC-derived IL-15-augmented MCAR-NKT cells (^PBMC15^MCAR-NKT) and (b) conventional MCAR-T cells (Fig. 2A to C and Fig. S2A and B). In this comparison, the ^PBMC15^MCAR-NKT cells were engineered to express soluble IL-15, whereas the conventional CAR-T cells were not, reflecting their respective designs in clinical trials where both cell types have demonstrated safety and therapeutic potential [48,60]. Our goal was to evaluate how ^Allo15^MCAR-NKT cells compare with these clinically relevant therapeutic cell products.

We first performed gene expression profiling using scRNA-seq. UMAP analysis across all 3 samples revealed 5 distinct transcriptional clusters: Cluster 1 represents proliferating cells, cluster 2 represents effector cells, cluster 3 represents memory-like cells, cluster 4 represents exhausted cells, and cluster 5 represents resting cells (Fig. 2D and Table S1) [28,42]. Notably, ^Allo15^MCAR-NKT cells were enriched for proliferating and effector cell populations while exhibiting lower frequencies of memory, exhausted, and resting cell states (Fig. 2E and F). In comparison, ^PBMC15^MCAR-NKT cells showed increased exhausted cell representation, and conventional MCAR-T cells displayed a greater proportion of memory-like cells (Fig. 2E and F).

Violin plot analyses further demonstrated that ^Allo15^MCAR-NKT cells exhibit a mixed T/NK transcriptional profile (Fig. 2G). Among the 3 groups, ^Allo15^MCAR-NKT cells had the lowest expression of canonical T cell genes but the highest expression of NK-associated genes, suggesting a skew toward NK-like features, similar to other in vitro-differentiated lymphocytes with enhanced NK-mediated cytotoxicity (Fig. 2G) [44,61,62]. In contrast, conventional MCAR-T cells exhibited strong T cell signatures and minimal NK features, while ^PBMC15^MCAR-NKT cells showed an intermediate phenotype (Fig. 2G). Additionally, consistent with the UMAP-based clustering, ^Allo15^MCAR-NKT cells expressed the highest effector and proliferation-related gene signatures (Fig. 2H). In addition, heatmap analysis supported these observations, showing that ^Allo15^MCAR-NKT cells had lower expression of key T cell-related genes (e.g., *CD2*, *CD40LG*, *LAT*, *ITM2A*, *LCK*, and *GRAP2*) and higher expression of NK cell-associated genes (e.g., *NKG7*, *XCL1*, *GNLY*, *KLRD1*, *CD226*, *CDK1*, and *NCAM1*), as well as proliferation markers (e.g., *MKI67*, *STMN1*, *LMNB1*, *PTMA*, *TMPO*, *VIM*, *ACTB*, and *TYMS*) and effector molecules (e.g., *GZMB*, *CCL5*, *FLNA*, *PRF1*, *MYO1G*, and *PIM1*) (Fig. 2I).

Additionally, a complete analysis of differentially expressed genes (DEGs) among the 3 therapeutic cell types revealed distinct, group-specific transcriptional signatures. ^Allo15^MCAR-NKT cells displayed an NK/innate-like cytotoxic and proliferative program, characterized by high expression of *GNLY*, *NCR3*, *NCAM1*, and cell cycle regulators *MKI67*, *TOP2A*, *CENPF*, and *STMN1* (Fig. S2C). In contrast, MCAR-T cells exhibited a transcriptional profile enriched for a naïve/central memory T cell program, marked by *CCR7*, *FOXP1*, *LRRN3*, *CD27*, and *CD2*, together with quiescence-associated genes *BTG1*, *TXNIP*, and *GIMAP4/7* (Fig. S2C). ^PBMC15^MCAR-NKT cells demonstrated an activation- and chemokine-driven immunoregulatory signature, including *CCL3*, *CCL4*, *IL2RA*, *GZMA*, *ENTPD1*, and the lineage-defining factor *ZBTB16*, along with myeloid-supporting cytokines *CSF2* and *CSF1* (Fig. S2C).

Pathway enrichment analysis revealed that, compared to ^PBMC15^MCAR-NKT cells, ^Allo15^MCAR-NKT cells up-regulated genes involved in T cell activation, migration, and cell division (Fig. 2J). Compared to conventional MCAR-T cells, ^Allo15^MCAR-NKT cells showed enrichment in pathways related to cell proliferation, granzyme B production, and NKT cell differentiation (Fig. 2J). Overall, these transcriptomic data suggest that ^Allo15^MCAR-NKT cells possess a unique hybrid T and NK identity and exhibit a highly cytotoxic, proliferative, and effector-primed transcriptional profile, supporting their potential as a potent immunotherapeutic platform.

We next validated these findings at the protein level using flow cytometry. Compared to ^PBMC15^MCAR-NKT and conventional MCAR-T cells, ^Allo15^MCAR-NKT cells expressed higher levels of NKRs, including CD56, CD161, NKG2D, DNAM-1, NKp44, and NKp30 (Fig. 2K and Fig. S2D). Intracellular staining further validated the functional competence of ^Allo15^MCAR-NKT cells, revealing elevated secretion of proinflammatory cytokines [interferon (IFN-γ), tumor necrosis factor-α (TNF-α), and IL-2] along with increased levels of cytotoxic effector molecules such as perforin and granzyme B, indicative of potent antitumor activity (Fig. 2L and Fig. S2E). These results collectively support that ^Allo15^MCAR-NKT cells are highly functional, with enhanced effector and cytolytic capacities, further validating their therapeutic potential against solid tumors such as MPM.

### ^Allo15^MCAR-NKT cells exhibit enhanced NK-like cytotoxicity compared to primary NK cells

Given the high expression of NKRs and pronounced NK-like features observed in ^Allo15^MCAR-NKT cells, we directly compared their intrinsic NK-like cytotoxicity to that of healthy donor PBMC-derived primary NK cells (Fig. 2A to C and Fig. S3A).

Transcriptomic profiling revealed that ^Allo15^MCAR-NKT cells, as expected, expressed higher levels of T cell-related genes and lower levels of NK lineage genes compared to primary NK cells, reflecting their inherent hybrid T/NK identity (Fig. S3B). Importantly, ^Allo15^MCAR-NKT cells also displayed elevated expression of proliferation-associated genes and memory-related signatures, alongside reduced expression of exhaustion markers (Fig. S3C). Specifically, both cell types expressed comparable levels of *ZBTB16* (encoding PLZF), a key transcription factor shared by NK and NKT lineages (Fig. S3D) [63–66]. However, ^Allo15^MCAR-NKT cells exhibited increased expression of activation markers such as *CD69* (encoding CD69), proliferation genes (e.g., *PCNA* encoding proliferating cell nuclear antigen and *MKI67* encoding Ki-67), memory-associated genes (e.g., *BCL2* encoding B cell lymphoma 2 and *XCL1* encoding lymphotactin XCL1), and activating NKRs including *NCR3* (encoding NKp30) and *CD226* (encoding DNAM-1) (Fig. S3D). Conversely, they expressed lower levels of exhaustion-associated genes such as *PRDM1* (encoding BLIMP-1) and *TIGIT* (encoding TIGIT) (Fig. S3D). These findings suggest that ^Allo15^MCAR-NKT cells exhibit a more activated and proliferative transcriptional profile, and are functionally primed for enhanced cytotoxic activity compared to primary NK cells.

Flow cytometric analyses further validated these results, demonstrating that ^Allo15^MCAR-NKT cells preferentially express activating NKRs, including NKG2D, DNAM-1, NKp44, and NKp30, while exhibiting little to no expression of inhibitory KIRs, a feature commonly associated with terminally differentiated NK cells (Fig. S3E and F) [67,68]. This profile suggests a bias toward enhanced cytotoxic responsiveness. This absence of inhibitory NKRs indicates a reduced threshold for activation, contributing to their enhanced cytotoxic potential. Moreover, ^Allo15^MCAR-NKT cells showed increased production of cytotoxic effector proteins, including perforin and granzyme B, further reinforcing their potent intrinsic cytolytic functionality (Fig. S3E and F).

To directly compare their antitumor activity, we evaluated the NKR-mediated cytotoxicity of ^Allo15^MCAR-NKT and NK cells against 4 tumor cell lines: MPM (H226), melanoma (A375), chronic myeloid leukemia (CML; K562), and ovarian cancer (OVCAR8) (Fig. S3G and H). To eliminate potential CAR-mediated effects, we used CRISPR-Cas9 to knock out *MSLN* in H226 and OVCAR8 cells, ensuring that all target tumor cells lacked MSLN expression. This allowed for a direct evaluation of NK-like, CAR-independent cytotoxicity. Each human tumor line was engineered to express a firefly luciferase and enhanced green florescence protein (EGFP) dual reporter (FG), enabling quantitative evaluation by flow cytometry through detection of GFP^+^ cells, as well as by bioluminescence-based assays such as luciferase activity measurements (Fig. S3G and H) [44].

In 24-h in vitro tumor cytotoxicity assays, ^Allo15^MCAR-NKT cells and primary NK cells exhibited dose-dependent killing activity as a function of the effector-to-target (E:T) ratio across multiple MSLN-deficient or low-expressing tumor cell lines, including H226 (MPM), A375 (melanoma), K562 (CML), and OVCAR8 (ovarian cancer) (Fig. S3H). However, ^Allo15^MCAR-NKT cells outperformed NK cells, exhibiting superior tumor cell lysis across all 4 tumor types, consistent with their enhanced activating NKR expression and CAR-independent intrinsic cytolytic profile (Fig. S3H).

To extend these findings in vivo, we developed a human ovarian cancer xenograft model in NSG mice using OVCAR8-FG^MSLN−/−^ cells, generated by CRISPR–Cas9-mediated deletion of the *MSLN* gene from the parental OVCAR8-FG line. The absence of MSLN expression on tumor cells ensured that cytotoxicity by therapeutic cells occurred in a CAR-independent manner, thereby enabling more accurate assessment and comparison of tumor cell killing mediated through intrinsic NKR pathways. Tumor engraftment was achieved through intraperitoneal injection to mimic peritoneal dissemination (Fig. S3I). Five days post-tumor inoculation, mice received a single intraperitoneal injection of either ^Allo15^MCAR-NKT cells or primary NK cells. Tumor burden was monitored via in vivo bioluminescence imaging (BLI) (Fig. S3I). While both cell types demonstrated tumor control, ^Allo15^MCAR-NKT cells exhibited superior antitumor efficacy, as evidenced by reduced tumor burden and extended overall survival (Fig. S3J to L).

Together, these results demonstrate that ^Allo15^MCAR-NKT cells possess greater intrinsic NK-like cytotoxicity than primary NK cells, both in vitro and in vivo, due to their heightened activation state, superior effector molecule production, and lack of inhibitory NKR expression, underscoring their therapeutic potential for targeting solid tumors through CAR-independent mechanisms.

Lastly, we recognize that a direct comparison between the 2 therapeutic cell types may not be optimal, particularly since the NK cells in the original experiments were not engineered with either CAR or IL-15. To address this, we generated healthy donor PBMC-derived IL-15-augmented, MCAR-armored NK (^PBMC15^MCAR-NK) cells using aAPC-mediated expansion and activation, achieving >95% NK cell purity and approximately 30% CAR transduction efficiency (Fig. S4A and B). These cells were then directly compared with ^Allo15^MCAR-NKT cells, with a primary focus on their tumor cell cytotoxic capacity. In the short-term in vitro tumor cell killing assay, both therapeutic cell products exhibited comparable and robust tumor cell killing against MSLN^+^ H226-FG human MPM cells and OVCAR8-FG human ovarian cancer cells (Fig. S4C and D). However, in the long-term in vitro serial tumor cell killing assay, ^Allo15^MCAR-NKT cells demonstrated superior tumor cell killing and more durable tumor control (Fig. S4E and F). The sustained antitumor activity of ^Allo15^MCAR-NKT cells compared to ^PBMC15^MCAR-NK cells is consistent with their prolonged survival and expansion in culture, as well as their maintained ability to produce effector cytokines, including IFN-γ and TNF-α (Fig. S4G and H). This advantage likely reflects the T cell-like immunobiology of invariant NKT cells, which exhibit stronger proliferative capacity and greater resistance to activation-induced cell death than NK cells [19,69,70].

### ^Allo15^MCAR-NKT cells demonstrate enhanced in vitro cytotoxicity against MPM tumor cells compared to ^PBMC15^MCAR-NKT and conventional MCAR-T cells

**Fig. 3.**
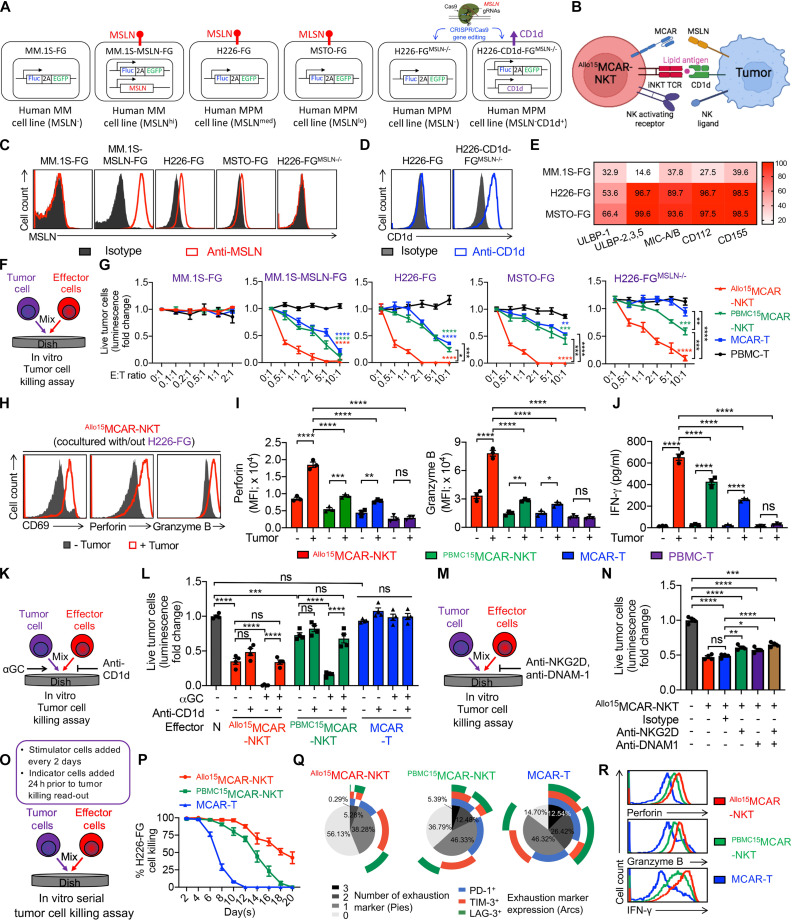
^Allo15^MCAR-NKT cells effectively kill MPM tumor cells, employing multiple targeting mechanisms while exhibiting low exhaustion characteristics. (A) Schematics showing the indicated human tumor cell lines. MM, multiple myeloma. (B) Diagram showing the tumor-targeting mechanisms of ^Allo15^MCAR-NKT cells. (C to E) FACS analyses of the expression of CAR target (C), TCR target (D), and NKR ligands (E) on tumor cells. The value in the heatmap represents the proportion of NKR ligand-expressing tumor cells. (F to J) Studying the antitumor capacity of ^Allo15^MCAR-NKT cells. (F) Experimental design. (G) Tumor cell killing data at 24 h (*n* = 4). (H) FACS detection of CD69, perforin, and granzyme B in ^Allo15^MCAR-NKT cells 24 h after coculture with H226-FG. (I) Quantification of (H) (*n* = 3). (J) ELISA quantification of IFN-γ production (*n* = 3). (K and L) Studying the tumor-targeting mechanism of ^Allo15^MCAR-NKT cells mediated by TCR. (K) Experimental design. (L) Tumor cell killing data at 24 h (E:T ratio = 2:1; *n* = 4 from 4 different cell product donors). (M and N) Studying the tumor-targeting mechanisms of ^Allo15^MCAR-NKT cells mediated by NKRs. (M) Experimental design. (N) Tumor cell killing data at 24 h (E:T ratio = 0.5:1; *n* = 4 from 4 different cell product donors). (O to R) Studying the long-term tumor killing capacity of ^Allo15^MCAR-NKT cells. (O) Experimental design. (P) Tumor cell killing data (*n* = 4). (Q) SPICE analysis of exhaustion marker expression on therapeutic cells collected on day 10. Pie charts reflect proportions of the indicated therapeutic cell groups expressing indicated numbers (0 to 3) of exhaustion markers. (R) FACS detection of intracellular markers in therapeutic cells collected on day 10. Representative of 3 experiments. Data are presented as the mean ± SEM. ns, not significant; *P < 0.05; **P < 0.01; ***P < 0.001; ****P < 0.0001 by one-way analysis of variance (ANOVA) (G, I, J, L, and N).

Due to the intrinsic expression of both NKRs and the invariant NKT TCR, ^Allo15^MCAR-NKT cells are capable of engaging multiple tumor cell targeting mechanisms, including CAR-, NKR-, and TCR-mediated recognition. To assess the in vitro cytotoxic potential and mechanisms of action (MOAs) of ^Allo15^MCAR-NKT cells, we utilized 6 tumor cell lines: 2 MM lines and 4 MPM lines (Fig. 3A to E). The MM.1S cell line, which expresses minimal NKR ligands and is resistant to NKR-mediated killing, was engineered to overexpress MSLN, creating MM.1S-MSLN cells, to evaluate CAR-specific cytotoxicity (Fig. 3A, C, and E). Among the MPM models, H226 and MSTO cell lines were selected based on differential MSLN expression—H226 with moderate and MSTO with low levels—allowing investigation across a spectrum of tumor antigen densities (Fig. 3C). To isolate CAR-independent mechanisms, *MSLN* was knocked out in H226 cells (H226-FG^MSLN−/−^), and to explore TCR-dependent killing, CD1d was overexpressed in these MSLN-deficient cells (H226-CD1d-FG^MSLN−/−^), facilitating NKT TCR engagement (Fig. 3C and D). All 6 tumor lines were transduced with FG, enabling both luminescence-based and flow cytometry quantification of tumor cell killing (Fig. 3A).

In 24-h coculture cytotoxicity assays, non-engineered PBMC-derived T (PBMC-T) cells showed no killing due to the lack of tumor-specific antigen recognition (Fig. 3F and G). Upon CAR engineering, MCAR-T cells selectively eliminated MSLN^+^ tumor cells (e.g., MM.1S-MSLN-FG and H226-FG) but not MSLN^−^ lines (e.g., MM.1S-FG and H226-FG^MSLN−/−^) (Fig. 3C and D). ^PBMC15^MCAR-NKT cells showed comparable killing of MSLN^+^ tumor cells; however, they also killed H226-FG^MSLN−/−^ cells, consistent with their endogenous NKR-mediated cytotoxicity (Fig. 3G) [20,24,25]. ^Allo15^MCAR-NKT cells exhibited the most potent killing across MSLN^+^ and MSLN^−^ tumor cells, confirming their engagement of both CAR-dependent and CAR-independent mechanisms (Fig. 3G). This superior cytotoxicity of ^Allo15^MCAR-NKT was associated with their elevated expression of the activation marker (i.e., CD69), enhanced secretion of cytotoxic effector molecules (i.e., perforin and granzyme B), and increased production of proinflammatory cytokines (i.e., IFN-γ) (Fig. 3H to J).

Notably, both ^Allo15^MCAR-NKT and ^PBMC15^MCAR-NKT cells were capable of mediating cytotoxicity against CD1d^+^ tumor cells. This effect was further potentiated by the addition of α-GC, a glycolipid antigen and potent invariant NKT cell agonist derived from marine sponge-associated bacteria, supporting a functional role for NKT TCR engagement in tumor recognition and killing (Fig. 3K and L). Blocking assays targeting NKRs (e.g., NKG2D and DNAM-1) attenuated tumor cell killing by ^Allo15^MCAR-NKT cells, confirming the importance of NKR-mediated mechanisms in their cytotoxic activity (Fig. 3M and N and Fig. S4I and J). Collectively, these results indicate that ^Allo15^MCAR-NKT cells engage in triple tumor targeting via CAR, NKR, and TCR pathways, offering superior tumor cell clearance over existing PBMC-derived therapeutic cells.

To assess durability, we performed a long-term serial tumor cell killing assay (Fig. 3O). While all engineered cell types effectively eliminated tumor cells initially, MCAR-T cells rapidly lost cytotoxic function, followed by ^PBMC15^MCAR-NKT cells (Fig. 3P). In contrast, ^Allo15^MCAR-NKT cells maintained prolonged killing capacity, consistent with their sustained expression of cytotoxic molecules (perforin and granzyme B), effector cytokines (IFN-γ), and low expression of T cell exhaustion markers (PD-1, TIM-3, and LAG-3) (Fig. 3Q and R). These findings highlight the rejuvenated potential of ^Allo15^MCAR-NKT cells as a durable and multifaceted immunotherapeutic platform capable of overcoming key limitations of current CAR-based strategies, such as restricted antigen specificity, limited persistence, and tumor immune evasion [17,71–73].

### ^Allo15^MCAR-NKT cells exhibit low expression of immune checkpoint molecules, associated with both epigenetic and signaling regulation

The low expression of exhaustion markers, many of which are immune checkpoint molecules, on ^Allo15^MCAR-NKT cells is a notable and potentially advantageous feature. This phenotype suggests reduced activation-induced dysfunction and enhanced functional persistence, supporting the hypothesis that these cells retain a “rejuvenated” capacity even under sustained antigen exposure [74–76]. To further investigate this phenomenon and its underlying mechanisms, we performed a series of functional and molecular analyses.

**Fig. 4.**
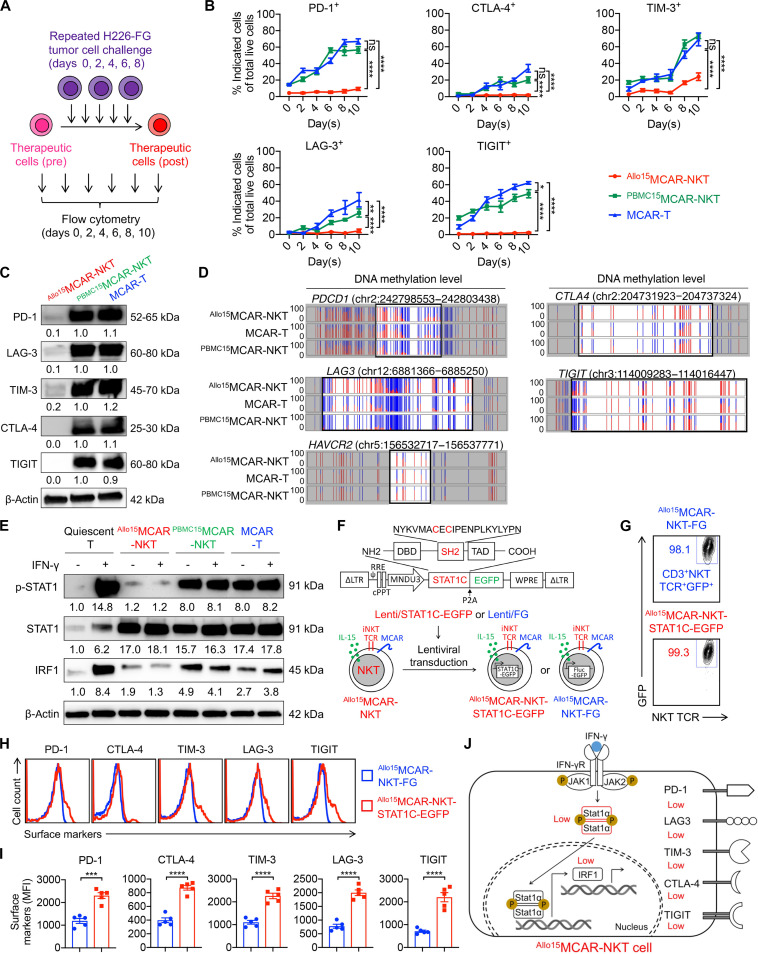
^Allo15^MCAR-NKT cells exhibit stably low expression of immune checkpoint molecules, associated with both epigenetic and signaling regulation. (A) Experimental design to evaluate immune checkpoint expression on therapeutic cells using a repeated tumor challenge assay. (B) FACS analyses of immune checkpoint expression on therapeutic cells over time (*n* = 4; *n* indicates different CB or PBMC donors). (C) Western blot analysis of immune checkpoints in therapeutic cells collected on day 10. (D) Methyl-seq analysis of the 3 therapeutic cell products. Vertical lines denote individual CpG sites, wherein red signifies methylation and blue signifies unmethylation. Differentially methylated regions (DMRs) are framed with black boxes. (E) Western blot analysis of key molecules involved in the IFN-𝛾 signaling pathway in the indicated therapeutic cells. (F to I) Rescue experiment by overexpressing a constitutively active STAT1 (STAT1C) in ^Allo15^MCAR-NKT cells. (F) Experimental design. (G) FACS detection of ^Allo15^MCAR-NKT-FG and ^Allo15^MCAR-NKT-STAT1C-EGFP cells. (H) FACS analyses of immune checkpoint expression on the indicated engineered ^Allo15^MCAR-NKT cells. (I) Quantification of (H) (*n* = 5). (J) Schematic illustrating a desensitized IFN-γ signaling network within ^Allo15^MCAR-NKT cells, which contributes to the distinctive “low immune checkpoint" phenotype exhibited by these cells. Representative of 1 (D) and 3 (A to C and E to I) experiments. Data are presented as the mean ± SEM. ns, not significant; *P < 0.05; **P < 0.01; ***P < 0.001; ****P < 0.0001 by Student’s *t* test (I) or 2-way ANOVA (B).

We first established a repeated tumor cell challenge assay, wherein H226-FG tumor cells were added to the culture every 2 d to simulate chronic antigen exposure (Fig. 4A). Therapeutic cells were then assessed for immune checkpoint molecule expression over time. ^PBMC15^MCAR-NKT cells and conventional MCAR-T cells showed progressive up-regulation of immune checkpoint molecules during repeated tumor challenge, including PD-1, CTLA-4, LAG-3, and TIGIT (Fig. 4B). Notably, TIM-3 expression remained relatively stable during the early phase but increased substantially during later stages (Fig. 4B), consistent with terminal exhaustion [77,78]. In contrast, ^Allo15^MCAR-NKT cells maintained markedly lower levels of these immune checkpoint molecules throughout the assay (Fig. 4B). These cells exhibited minimal surface expression of PD-1, CTLA-4, LAG-3, and TIGIT across time points, and although TIM-3 was up-regulated at the terminal stage, its levels remained significantly lower than in comparator cell types (Fig. 4B). Western blot analysis further confirmed the reduced expression of these immune checkpoints at the protein level in ^Allo15^MCAR-NKT cells during late-stage coculture (Fig. 4C).

To explore the regulatory mechanisms underlying this unique phenotype, we assessed both epigenetic modifications and key signaling pathways. Methylation sequencing (Methyl-seq) revealed hypermethylation in the promoter regions of the 5 checkpoint genes (i.e., *PDCD1*, *LAG3*, *HAVCR2*, *CTLA4*, and *TIGIT*) in ^Allo15^MCAR-NKT cells, suggesting that epigenetic silencing may contribute to their low expression (Fig. 4D). We next examined the IFN-γ–Janus kinase (JAK)–signal transducer and activator of transcription 1 (STAT1) signaling pathway, a central regulator of immune checkpoint gene transcription [79]. ^Allo15^MCAR-NKT cells displayed markedly reduced STAT1 phosphorylation at baseline and upon IFN-γ stimulation, whereas ^PBMC15^MCAR-NKT and MCAR-T cells exhibited significantly elevated phosphorylation levels, including under unstimulated conditions, suggesting differential activation states (Fig. 4E). Furthermore, expression of IFN-γ-inducible transcription factors, such as IRF1, was also reduced, indicating a blunted transcriptional response to inflammatory cues (Fig. 4E). To further assess the contribution of STAT1 signaling, we overexpressed a constitutively active STAT1 construct in ^Allo15^MCAR-NKT cells (Fig. 4F and G) [80]. Enforced STAT1 activation led to increased expression of multiple immune checkpoint molecules, including PD-1, CTLA-4, LAG-3, TIM-3, and TIGIT (Fig. 4H and I), indicating that attenuated STAT1 signaling contributes to their reduced baseline expression (Fig. 4J). Future studies will further clarify the role of epigenetic modifications, for example, through targeted demethylation approaches.

Together, these data reveal a stably maintained “low immune checkpoint” phenotype in ^Allo15^MCAR-NKT cells, governed by both epigenetic and signaling-level regulation. Importantly, this phenotype persists even under inflammatory conditions and repeated tumor antigen exposure, potentially conferring superior functional persistence and reduced exhaustion. These properties underscore the therapeutic promise of ^Allo15^MCAR-NKT cells as a resilient and rejuvenated cellular platform for the treatment of MPM and potentially other solid tumors.

### ^Allo15^MCAR-NKT cells exhibit robust in vivo tumor control in subcutaneous MPM xenograft models, with low expression of immune checkpoints and rejuvenated potential

**Fig. 5.**
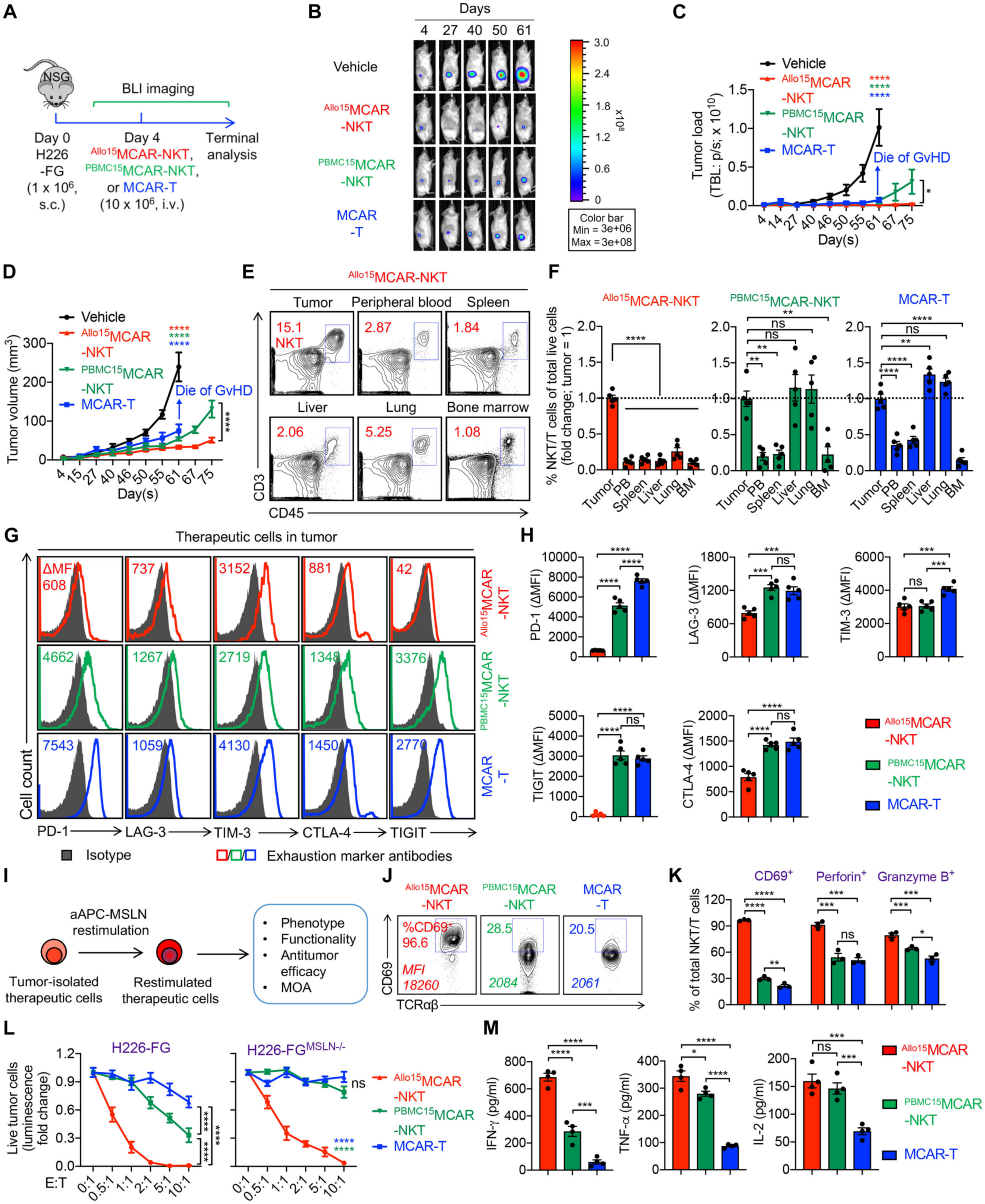
^Allo15^MCAR-NKT cells exhibit potent in vivo killing of MPM tumor cells, with low expression of immune checkpoints and rejuvenated potential. (A to H) Studying the in vivo antitumor efficacy of ^Allo15^MCAR-NKT cells in an H226-FG subcutaneous (s.c.) human MPM xenograft NSG mouse model. (A) Experimental design. BLI, live animal bioluminescence imaging; i.v., intravenous. (B) BLI images measuring tumor loads in experimental mice over time. (C) Quantification of (B) (*n* = 5). GvHD, graft-versus-host disease. (D) Tumor volume measured in experimental mice over time (*n* = 5). (E) FACS detection of ^Allo15^MCAR-NKT cells in various tissues of experimental mice terminated on day 60. (F) FACS analyses of tissue biodistribution of the 3 therapeutic cells in experimental mice terminated on day 60 (*n* = 5). The percentage of therapeutic cells in the tumor is normalized to 1. (G) FACS detection of expression of immune checkpoints on the indicated therapeutic cells. The ΔMFI is defined as the mean florescence intensity (MFI) of stained samples minus that of isotype controls in the FACS plots. (H) Quantification of (G) (*n* = 5). (I to M) Studying the rejuvenated potential of in vivo tumor-isolated and restimulated ^Allo15^MCAR-NKT cells. (I) Experimental design. Therapeutic cells isolated from tumors of experimental mice terminated on day 60 were used for the assay. (J) FACS detection of surface expression of T cell activation marker CD69 on the indicated therapeutic cells. (K) Percentage of CD69^+^, perforin^+^, and granzyme B^+^ cell population among the total therapeutic cells (*n* = 3). (L) Tumor cell killing data by restimulated therapeutic cells at 24 h (*n* = 4). (M) ELISA analyses of IFN-γ, TNF-α, and IL-2 secretion by restimulated therapeutic cells at 24 h (*n* = 4). Representative of 2 experiments. Data are presented as the mean ± SEM. ns, not significant; *P < 0.05; **P < 0.01; ***P < 0.001; ****P < 0.0001 by one-way ANOVA.

The in vivo tumor killing capacity of ^Allo15^MCAR-NKT cells was evaluated utilizing an H226-FG subcutaneous human MPM xenograft NSG mouse model (Fig. 5A). ^PBMC15^MCAR-NKT and conventional MCAR-T cells were included as the benchmark controls. Both BLI and measurements of tumor size showed marked reductions in tumor burden with all 3 therapeutic cell products. Notably, ^Allo15^MCAR-NKT cells exhibited enhanced tumor suppression compared to ^PBMC15^MCAR-NKT and MCAR-T cells (Fig. 5B to D and Fig. S5A). Additionally, MCAR-T cells resulted in mouse mortality due to GvHD, whereas both HSPC- and PBMC-derived MCAR-NKT cell products did not induce GvHD (Fig. 5B to D and Fig. S5A), consistent with previous preclinical and clinical findings showing minimal GvHD risk with NKT cells [81–84].

Flow cytometry analysis of various tissues on day 60 post-treatment revealed that ^Allo15^MCAR-NKT cells were broadly distributed across tumors, peripheral blood, spleen, lung, and bone marrow, with the highest frequency observed in tumor tissues (Fig. 5E and F). In contrast, both ^PBMC15^MCAR-NKT and MCAR-T cells were not only present in the tumor but also enriched in nontarget tissues such as the liver and lung (Fig. 5F and Fig. S5B). Notably, MCAR-T cells exhibited disproportionately high accumulation in the liver (up to 60%) and lung (over 50%), suggesting the onset of systemic GvHD (Fig. 5F and Fig. S5B) [85,86]. These in vivo results collectively demonstrate that ^Allo15^MCAR-NKT cells possess robust tumor-infiltrating capacity with minimal off-target accumulation.

Notably, the dosing strategy used in preclinical models is an important factor in interpreting both efficacy and safety outcomes. Higher cell doses may accelerate and exacerbate xenogeneic GvHD in the MCAR-T cell group, thereby complicating direct comparisons with ^Allo15^MCAR-NKT and ^PBMC15^MCAR-NKT cells. To address this, we performed additional in vivo studies using lower and more clinically relevant doses (1 × 10^6^ and 3 × 10^6^ CAR^+^ cells per mouse) (Fig. S5C). Under both conditions, ^Allo15^MCAR-NKT cells demonstrated superior tumor control, as evidenced by slower tumor growth and improved tumor suppression through day 35 (Fig. S5D to F). These findings indicate that the enhanced antitumor activity and favorable safety profile of ^Allo15^MCAR-NKT cells are maintained across dose levels. Nevertheless, dose-dependent effects on cell expansion, persistence, and toxicity may still influence therapeutic outcomes. Further studies in models that more closely recapitulate human pharmacokinetics and immune interactions will be important to refine dose translation and optimize clinical applicability.

To further evaluate the functional status of tumor-infiltrating therapeutic cells, we analyzed the expression of immune checkpoint molecules. ^Allo15^MCAR-NKT cells displayed markedly lower expression of inhibitory receptors including PD-1, LAG-3, TIM-3, TIGIT, and CTLA-4 compared to ^PBMC15^MCAR-NKT and MCAR-T cells (Fig. 5G and H), indicating a less exhausted, more functionally active state. Since these immune checkpoints are also markers of T cell exhaustion, their reduced expression suggests sustained effector potential in the TME [74,87,88]. Taken together, the enhanced antitumor efficacy of ^Allo15^MCAR-NKT cells can be attributed to their potent tumor homing and infiltration, and maintenance of a low-exhaustion, high-function phenotype, supporting their promise as a potent allogeneic cell therapy for treating MPM.

We next investigated the revitalization potential of ^Allo15^MCAR-NKT cells by isolating these cells from tumors in vivo and subjecting them to restimulation to evaluate their functional capacity following exposure to the MPM TME (Fig. 5I). Interestingly, restimulated ^Allo15^MCAR-NKT cells demonstrated superior effector function compared to ^PBMC15^MCAR-NKT and MCAR-T cells, as reflected by elevated levels of activation marker CD69 and cytotoxic mediators such as perforin and granzyme B, suggesting enhanced functional durability (Fig. 5J and K and Fig. S5G). These cells also maintained superior tumor-killing capacity and robust cytokine production, including IFN-γ, TNF-α, and IL-2 (Fig. 5L and M). Overall, these results indicate that ^Allo15^MCAR-NKT cells possess a durable effector phenotype and maintain functional resilience after tumor engagement, underscoring their capacity to resist functional exhaustion and sustain antitumor activity within the suppressive TME.

### ^Allo15^MCAR-NKT cells demonstrate potent in vivo killing against metastatic MPM tumor cells, which can be further potentiated by anti-TIM-3 checkpoint blockade

**Fig. 6.**
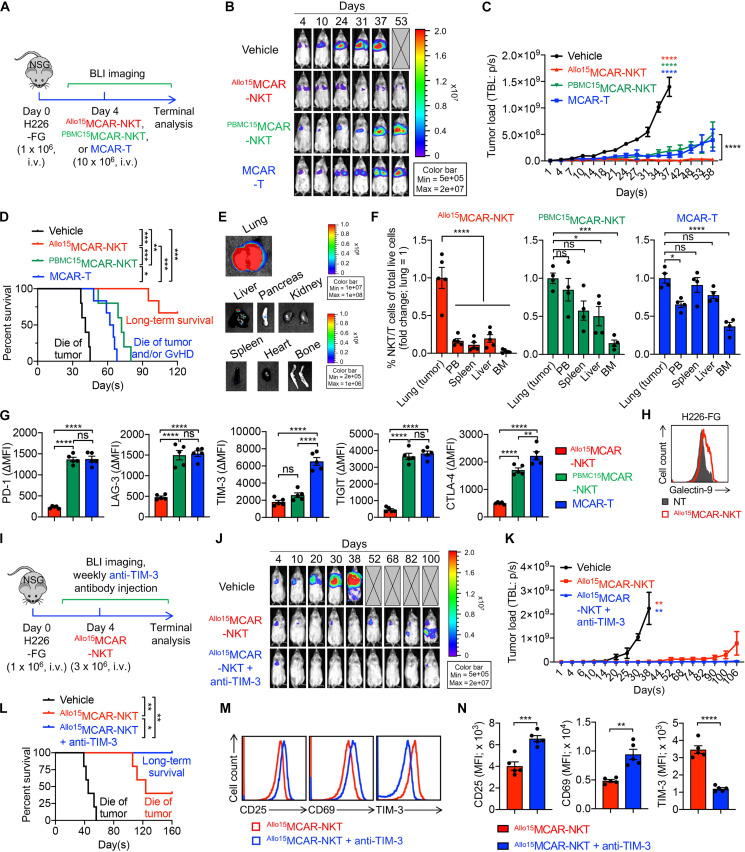
^Allo15^MCAR-NKT cells demonstrate potent in vivo killing against metastatic MPM tumor cells, which can be further potentiated by anti-TIM-3 checkpoint blockade. (A to H) Studying the in vivo antitumor efficacy of ^Allo15^MCAR-NKT cells in an H226-FG metastatic human MPM xenograft NSG mouse model. (A) Experimental design. (B) BLI images measuring tumor loads in experimental mice over time. (C) Quantification of (B) (*n* = 5 to 6). (D) Kaplan–Meier survival curves of experimental mice over time (*n* = 5 to 6). (E) Tissue BLI images showing the H226-FG tumor cell biodistribution. (F) FACS analyses of tissue biodistribution of the 3 therapeutic cells in experimental mice terminated on day 50 (*n* = 4 to 5). The percentage of therapeutic cells in the lung (tumor) is normalized to 1. (G) FACS detection of expression of immune checkpoints on the indicated therapeutic cells (*n* = 5). (H) FACS detection of expression of galectin-9, a TIM-3-binding ligand, on H226-FG tumor cells isolated from the lungs of experimental mice. (I to N) Studying the impact of TIM-3 blockade on antitumor efficacy of ^Allo15^MCAR-NKT cells in an H226-FG metastatic human MPM xenograft NSG mouse model. (I) Experimental design. (J) BLI images measuring tumor loads in experimental mice over time. (K) Quantification of (J) (*n* = 5). (D) Kaplan–Meier survival curves of experimental mice over time (*n* = 5). (M) FACS detection of expression of T cell activation markers (i.e., CD25 and CD69) and TIM-3 on ^Allo15^MCAR-NKT cells. (N) Quantification of (M) (*n* = 5). Representative of 2 experiments. Data are presented as the mean ± SEM. ns, not significant; *P < 0.05; **P < 0.01; ***P < 0.001; ****P < 0.0001 by Student’s *t* test (N), one-way ANOVA (C, F, G, and K), or log rank (Mantel–Cox) test adjusted for multiple comparisons (D and L).

Given that MPM tumor cells frequently infiltrate the lungs and surrounding thoracic structures, we established 2 metastatic MPM xenograft mouse model via intravenous injection of H226-FG or MSTO-FG tumor cells (Fig. 6A to D and I to L and Fig. S6A to F). These models closely mimic the metastatic progression and anatomical localization of human MPM, as the tumor cells preferentially grow in the lungs and ultimately lead to mouse mortality. Following tumor inoculation, mice received intravenous administration of 3 different therapeutic cell types. In the H226-FG model, while all 3 therapies demonstrated some degree of tumor control, ^Allo15^MCAR-NKT cells exhibited the most potent antitumor activity, resulting in reduced MPM tumor burden and prolonged mouse survival (Fig. 6B to D). Similarly, in the MSTO-FG model, ^Allo15^MCAR-NKT cells demonstrated robust tumor control, with some mice achieving early tumor clearance during the course of the experiment (Fig. S6B and C). Of note, in some cases, ^Allo15^MCAR-NKT cells were unable to completely eradicate tumor cells, resulting in relapse and subsequent mortality (Fig. 6B to D and Fig. S6B and C). However, given the allogeneic, off-the-shelf nature of this platform, multiple or repeated dosing is feasible and may provide additional therapeutic benefit in hard-to-treat and relapsed MPM.

The 3 therapeutic cell types demonstrated distinct in vivo kinetic profiles. In the H226-FG xenograft mouse model, ^Allo15^MCAR-NKT cells expanded in vivo for up to 3 weeks, followed by a gradual decline, and were undetectable by 8 weeks post-injection, indicating a robust expansion capacity without uncontrolled long-term persistence (Fig. S7A to C). PBMC-derived ^PBMC15^MCAR-NKT cells exhibited a similar pattern, expanding during the first 2 weeks, then declining to a stable level and persisting for up to 10 weeks until mice succumbed to tumor progression (Fig. S7A to C). In contrast, conventional MCAR-T cells displayed stable levels for the first 4 weeks, followed by a marked expansion reaching over 40% of circulating lymphocytes by week 7, which was associated with severe xenogeneic GvHD and subsequent mortality (Fig. S7A to C). These findings suggest that ^Allo15^MCAR-NKT cells combine potent in vivo expansion with a self-limiting persistence profile, potentially minimizing long-term safety risks compared to conventional MCAR-T cells.

Given the lung-localized tumor growth, we observed that ^Allo15^MCAR-NKT cells preferentially infiltrated into the lungs, whereas ^PBMC15^MCAR-NKT and MCAR-T cells, despite also accumulating in the lungs, were more broadly distributed across multiple tissues, including blood, spleen, liver, and bone marrow (Fig. 6E and F and Figs. S6F and S7D). Immunohistochemical analysis further demonstrated that ^Allo15^MCAR-NKT cell-treated mice harbored markedly fewer GFP^+^ tumor cells in the lungs, consistent with BLI results (Fig. S6F). In addition, compared with conventional MCAR-T and ^PBMC15^MCAR-NKT cells, ^Allo15^MCAR-NKT cells showed superior persistence within lung tissue and enhanced infiltration into tumor nodules, supporting their potent antitumor activity (Fig. S6F). Collectively, these findings highlight the enhanced tumor homing and infiltration capabilities of ^Allo15^MCAR-NKT cells, regardless of whether the tumor is a primary solid mass (Fig. 5F) or has metastasized to the lung (Fig. 6F). The superior tumor-homing capacity of ^Allo15^MCAR-NKT cells may be attributable to their elevated expression of tissue inflammation-associated chemokine receptors, including CCR1, CCR5, CXCR3, and CXCR6 (Fig. S7E).

Flow cytometric analysis of immune checkpoint expression mirrored the results observed in the subcutaneous tumor model (Fig. 5G and H). Notably, ^Allo15^MCAR-NKT cells exhibited significantly reduced levels of exhaustion-associated and inhibitory receptors, including PD-1, CTLA-4, TIGIT, TIM-3, and LAG-3, reinforcing their rejuvenated and functionally competent phenotype (Fig. 6G and Figs. S6D and S7F).

Notably, ^Allo15^MCAR-NKT cells exhibited low to minimal expression of PD-1, CTLA-4, TIGIT, and LAG-3, while these cells retained detectable levels of TIM-3, comparable to ^PBMC15^MCAR-NKT cells but lower than conventional MCAR-T cells (Fig. 6G and Figs. S6D and S7F). Previous studies have demonstrated that co-blockade of TIM-3 and PD-1 can reduce tumor progression and enhance antitumor T cell responses [89], suggesting that TIM-3 may serve as a key immune checkpoint even in cells with otherwise low exhaustion marker expression. Moreover, galectin-9, a known ligand for TIM-3, was expressed on the H226-FG tumor cells (Fig. 6H), suggesting a potential immune evasion mechanism [89,90].

To explore whether TIM-3 blockade could further potentiate ^Allo15^MCAR-NKT cell function, we tested a combination therapy using ^Allo15^MCAR-NKT cells and an anti-TIM-3 antibody (sabatolimab biosimilar) in an H226-FG metastatic xenograft mouse model (Fig. 6I). Despite the already potent antitumor effects of ^Allo15^MCAR-NKT cells alone, combination therapy further enhanced their efficacy, leading to significantly improved tumor control and extended survival (Fig. 6J to L and Fig. S6E). Additionally, anti-TIM-3 treatment increased the expression of activation markers CD25 and CD69 on ^Allo15^MCAR-NKT cells and reduced TIM-3 expression, indicating effective checkpoint inhibition (Fig. 6M and N).

Overall, these results suggest that ^Allo15^MCAR-NKT cells already exhibit potent antitumor activity against metastatic MPM and maintain a rejuvenated functional phenotype. Importantly, TIM-3 blockade synergizes with ^Allo15^MCAR-NKT cell therapy to overcome residual immunosuppression and further enhance antitumor responses, supporting the feasibility and rationale for combining checkpoint inhibition with allogeneic CAR-NKT cell therapy to improve clinical efficacy in the treatment of MPM.

### ^Allo15^MCAR-NKT cells remodel the MPM TME by targeting CD1d^+^ immunosuppressive cells while maintaining a rejuvenated phenotype

**Fig. 7.**
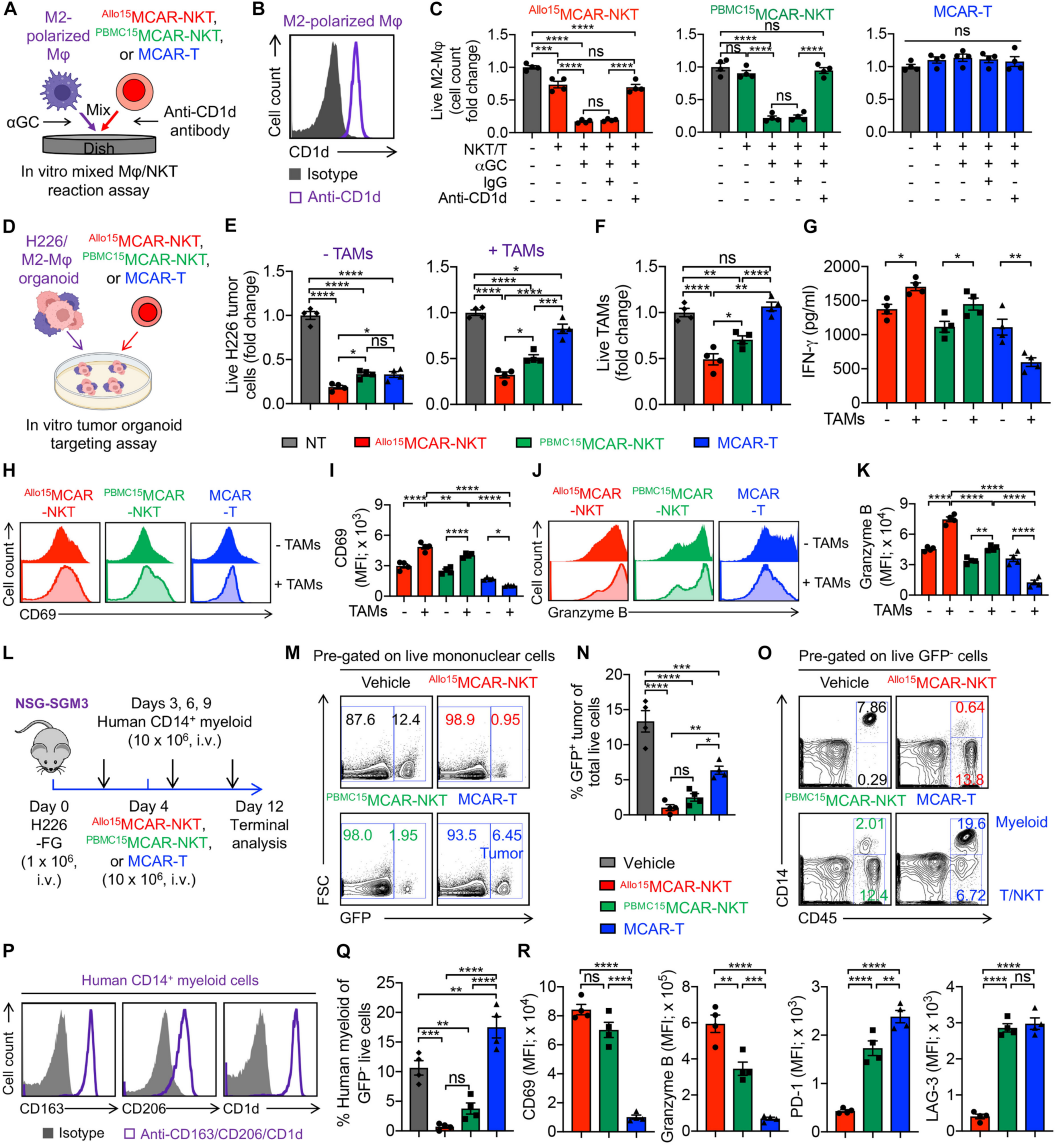
^Allo15^MCAR-NKT cells alter the MPM TME by targeting CD1d^+^ immunosuppressive cells. (A to C) Studying ^Allo15^MCAR-NKT cell targeting of human M2-polarized macrophages (Mφs). (A) Experimental design. (B) FACS detection of CD1d expression on Mφs. (C) Mφ killing data at 24 h (*n* = 4). (D to K) Studying ^Allo15^MCAR-NKT cell targeting of MPM TME using H226/Mφ coculture organoids. (D) Experimental design. (E) H226 tumor cell killing data at 24 h (*n* = 4). (F) TAM killing data at 24 h (*n* = 4). (G) ELISA quantification of IFN-γ production (*n* = 4). (H) FACS detection of surface activation marker CD69 expression in the indicated therapeutic cells. (I) Quantification of (H) (*n* = 4). (J) FACS detection of intracellular cytotoxic molecule granzyme B production in the indicated therapeutic cells. (K) Quantification of (J) (*n* = 4). (L to R) Studying ^Allo15^MCAR-NKT cell targeting of MPM TME using an H226-FG human MPM xenograft NSG-SGM3 mouse model. (L) Experimental design. Human CD14^+^ myeloid cells were isolated via MACS sorting and injected into experimental mice on days 3, 6, and 9 to establish macrophage populations within the MPM TME. (M) FACS plots showing the presence of H226-FG tumor cells in mouse lungs. (N) Quantification of (M) (*n* = 4). (O) FACS plots showing the presence of human myeloid and T/NKT cells in mouse lungs. (P) FACS plots showing the expression of human CD163, CD206, and CD1d on myeloid cells isolated from the lungs of experimental mice without therapeutic cell treatment. (Q) Quantification of (O) (*n* = 4). (R) FACS detection of expression of CD69, granzyme B, PD-1, and LAG-3 on the indicated therapeutic cells (*n* = 4). Representative of 2 (L to R) and 3 (A to K) experiments. Data are presented as the mean ± SEM. ns, not significant; *P < 0.05; **P < 0.01; ***P < 0.001; ****P < 0.0001 by Student’s *t* test (G) or one-way ANOVA (C, E, F, I, K, N, Q, and R).

The TME is a critical driver of MPM progression, immune evasion, and resistance to therapy [91,92]. Notably, MPM is enriched with immunosuppressive cells, particularly tumor-associated macrophages (TAMs), which express high levels of CD1d, suggesting that they may be susceptible to targeting by NKT cells [93,94]. To evaluate the TME-modulating functions of ^Allo15^MCAR-NKT cells, we performed a series of complementary assays, including a 2-dimensional (2D) macrophage-killing assay (Fig. 7A to C), a 3D tumor/TAM organoid model (Fig. 7D to K), and a humanized MPM NSG-SGM3 xenograft model (Fig. 7L to R).

We first generated M2-polarized macrophages from healthy donor PBMCs using a protocol that induces hallmark immunosuppressive characteristics (Fig. 7A) [95,96]. These macrophages expressed high levels of CD1d, indicating their potential susceptibility to NKT TCR-mediated recognition (Fig. 7B). In direct coculture assays, both ^Allo15^MCAR-NKT and ^PBMC15^MCAR-NKT cells efficiently killed M2 macrophages in the presence of αGC, demonstrating CD1d-dependent cytotoxicity (Fig. 7C). This killing was abrogated by CD1d antibody blockade, further confirming pathway specificity (Fig. 7C). Interestingly, ^Allo15^MCAR-NKT cells retained partial macrophage-killing activity even without αGC stimulation, suggesting additional NKR-mediated cytotoxic mechanisms (Fig. 7C). Conventional MCAR-T cells did not kill M2 macrophages under any condition, indicating that TAMs are resistant to conventional T cell-based CAR therapies (Fig. 7C).

We next constructed 3D organoids composed of H226 tumor cells alone or a mixture of H226 cells and M2 macrophages to model TAM-rich tumor niches (Fig. 7D). In tumor-only organoids, all 3 therapeutic cell types displayed potent antitumor activity (Fig. 7E). However, in tumor/TAM organoids, MCAR-T cells exhibited markedly reduced tumor killing, diminished IFN-γ production, lower CD69 expression, and reduced granzyme B induction, which were consistent with functional suppression by TAMs (Fig. 7E to K) [95,97,98]. In contrast, both ^Allo15^MCAR-NKT and ^PBMC15^MCAR-NKT cells maintained robust tumor cytotoxicity and remained highly activated in the presence of TAMs, reflecting their ability to simultaneously target tumor cells and reshape the TAM compartment within the organoid microenvironment (Fig. 7E to K).

Finally, we evaluated TME remodeling in a human H226-FG MPM NSG-SGM3 model (Fig. 7L). NSG-SGM3 mice express human stem cell factor (SCF), granulocyte-macrophage colony-stimulating factor (GM-CSF), and IL-3, enabling enhanced engraftment and maintenance of human myeloid populations in vivo [99,100]. Following infusion of human myeloid cells, mice developed a TAM-rich, immunosuppressive TME in the lung (Fig. 7L to O). Treatment with ^Allo15^MCAR-NKT or ^PBMC15^MCAR-NKT cells resulted in potent tumor clearance, markedly surpassing the efficacy of MCAR-T cells (Fig. 7M and N). These in vivo results suggest that MCAR-T cell function is inhibited by the immunosuppressive TME, whereas MCAR-NKT cells are able to overcome this suppression and retain robust antitumor activity. Notably, TAMs in this model expressed high levels of CD163 and CD206, consistent with M2 polarization and their known clinical association with CAR-T cell resistance (Fig. 7O and P) [18,101].

^Allo15^MCAR-NKT and ^PBMC15^MCAR-NKT cell treatment markedly reduced the myeloid compartment, indicating active elimination of TAMs in vivo (Fig. 7O and Q). In contrast, MCAR-T cell-treated mice displayed an accumulation of myeloid cells (Fig. 7O and Q), suggesting that interactions between CAR-T cells and myeloid populations may drive myeloid expansion, exacerbate immunosuppression, and potentially contribute to adverse events such as cytokine release syndrome (CRS) [102–104]. Phenotypic profiling further revealed that ^Allo15^MCAR-NKT cells maintained high CD69 expression, strong granzyme B production, and lower PD-1 and LAG-3 expression within the TME, indicating that they preserve effector competence despite the immunosuppressive environment, in part by actively remodeling it (Fig. 7R).

In summary, ^Allo15^MCAR-NKT cells effectively target both MPM tumor cells and immunosuppressive CD1d^+^ myeloid cells, enabling robust antitumor activity even within a hostile TME. Their dual tumor and TME remodeling capacity, sustained effector function, and low exhaustion support their therapeutic potential in MPM.

### ^Allo15^MCAR-NKT cells exhibit a favorable safety profile, characterized by low GvHD risk, limited CRS toxicity, and minimal off-target organ damage

**Fig. 8.**
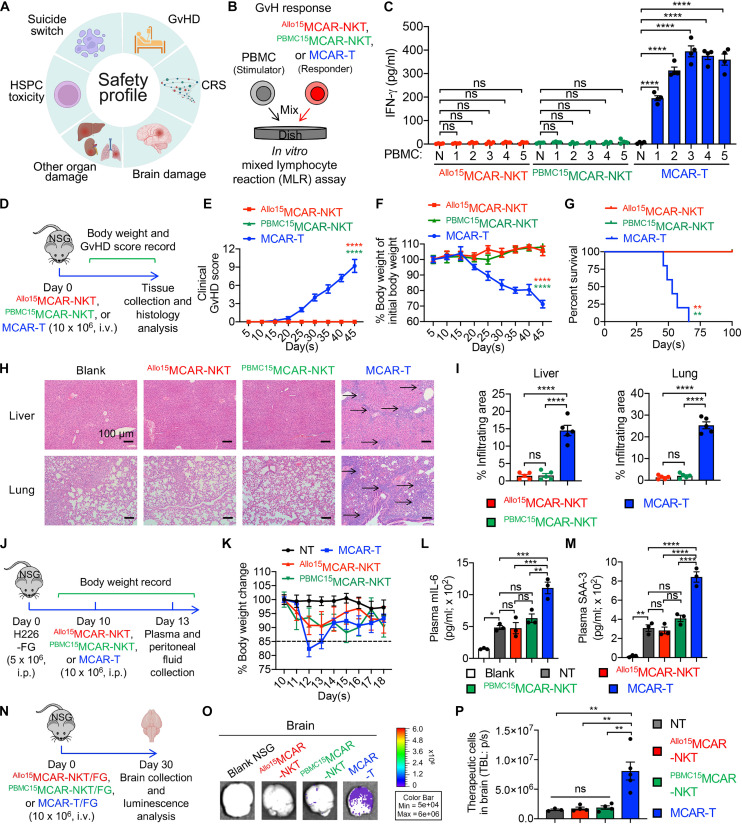
^Allo15^MCAR-NKT cells demonstrate high safety profile by exhibiting low GvHD risk, minimal CRS characteristics, and negligible organ damage. (A) Diagram showing the evaluation aspects of safety profile for ^Allo15^MCAR-NKT cells. Created in BioRender. LI, Y. (2025) https://BioRender.com/4h3j213. (B and C) Studying the graft-versus-host (GvH) response of ^Allo15^MCAR-NKT cells using an in vitro MLR assay. (B) Experimental design. (C) ELISA quantification of IFN-γ secretion on day 4 (*n* = 4). N, no addition of stimulators. (D to I) Studying the GvHD risk of ^Allo15^MCAR-NKT cells using a xenograft mouse model. (D) Experimental design. (E) Clinical GvHD score (*n* = 5). (F) Body weight change (*n* = 5). (G) Kaplan–Meier survival curves (*n* = 5). (H) H&E-stained tissue sections. Tissues were collected from ^Allo15^MCAR-NKT and ^PBMC15^MCAR-NKT cell-treated mice on day 60. For the MCAR-T group, mice were euthanized upon reaching predefined humane endpoints, and tissues were collected at those corresponding timepoints. The arrows point to mononuclear cell infiltrates. (I) Quantification of (H) (*n* = 5). (J to M). Studying CRS response induced by ^Allo15^MCAR-NKT cells using a xenograft mouse model. (J) Experimental design. (K) Body weight change (*n* = 3). (L and M) ELISA quantification of mouse IL-6 (L) and SAA-3 (M) secretion in mouse plasma collected on day 13 (*n* = 3). SAA-3, serum amyloid A-3. NT, plasma sample from tumor-bearing mice receiving no therapeutic cell treatment. (N to P) Studying the brain infiltration and neurotoxicity of ^Allo15^MCAR-NKT cells using a xenograft mouse model. (N) Experimental design. Therapeutic cells were labeled with the FG and injected into NSG mice to assess their infiltration into the mouse brain. (O) BLI images showing the presence of therapeutic cells in mouse brain collected on day 30. (P) Quantification of (O) (*n* = 3 to 5). Representative of 3 experiments. Data are presented as the mean ± SEM. ns, not significant; *P < 0.05; **P < 0.01; ***P < 0.001; ****P < 0.0001 by one-way ANOVA (C, E, F, I, L, M, and P) or by log-rank (Mantel–Cox) test adjusted for multiple comparisons (G).

We conducted a thorough assessment of the safety profile of ^Allo15^MCAR-NKT cells, focusing on 6 key parameters: (a) GvHD, (b) CRS, (c) neurotoxicity and brain infiltration, (d) systemic organ toxicity, (e) HSPC toxicity, and (f) incorporation of a suicide safety switch (Fig. 8A). These evaluations are critical to ensure the translational feasibility and clinical safety of allogeneic CAR-NKT cell therapy.

GvHD remains a major concern for allogeneic cell therapies due to recognition of host alloantigens by polymorphic TCRs [17,86,105,106]. However, invariant NKT cells utilize a semi-invariant TCR that recognizes the nonpolymorphic CD1d molecule, significantly reducing alloreactivity [41,82,83,107]. We first assessed GvHD potential using an in vitro mixed lymphocyte reaction (MLR) assay, in which irradiated healthy donor PBMCs were cocultured with the 3 therapeutic cell products. IFN-γ levels were measured on day 4 (Fig. 8B). Both ^Allo15^MCAR-NKT and ^PBMC15^MCAR-NKT cells failed to induce IFN-γ production, whereas conventional MCAR-T cells elicited strong IFN-γ secretion, indicating a higher risk of allo-reactivity and GvHD for the latter (Fig. 8C).

To confirm these findings in vivo, we employed 2 complementary mouse models: standard NSG mice and NSG mice deficient in both major histocompatibility complex (MHC) class I and class II (NSG-MHC I/II DKO) (Fig. 8D and Fig. S8A). The NSG-MHC I/II DKO model lacks expression of MHC class I molecules (H2-K and H2-D) and MHC class II molecules (I-A), resulting in a delayed onset and reduced severity of GvHD [108]. These 2 models are therefore well suited for investigating the in vivo mechanisms of xenogeneic GvHD. By administering the 3 therapeutic cell products across both models, we were able to delineate and compare the GvHD-inducing potential of each cell type. In standard NSG mice, treatment with conventional MCAR-T cells resulted in rapid and lethal xenogeneic GvHD, characterized by marked weight loss, elevated clinical GvHD scores (assessed based on body weight, activity, posture, skin integrity, diarrhea, and fur condition), and extensive immune cell infiltration in vital organs such as the liver and lungs, ultimately leading to mortality (Fig. 8E to I). These symptoms were markedly attenuated and delayed in NSG-MHC I/II DKO mice, indicating that MHC-dependent interactions play a critical role in the onset and severity of xenogeneic GvHD (Fig. S8B to D). In contrast, treatment with either ^Allo15^MCAR-NKT or ^PBMC15^MCAR-NKT cells in both mouse models did not result in detectable clinical signs of GvHD. Mice maintained stable body weight and demonstrated prolonged survival, supporting a favorable safety profile with minimal alloreactive potential and reduced risk of GvHD (Fig. 8E to I and Fig. S8B to D).

CRS is a life-threatening toxicity associated with CAR-T cell therapies, characterized by systemic inflammation driven by rapid immune activation and the release of high levels of proinflammatory cytokines such as IL-6, IFN-γ, and TNF-α [6,98]. In an H226-FG MPM xenograft model, all 3 therapeutic cells demonstrated robust tumor-killing activity (Fig. S8E and F); however, conventional MCAR-T cells triggered pronounced CRS, evidenced by marked body weight loss and elevated serum mouse serum amyloid A3 (SAA-3) and IL-6 levels (Fig. 8J to M and Fig. S9A). In contrast, treatment with ^Allo15^MCAR-NKT or ^PBMC15^MCAR-NKT cells did not induce body weight loss or CRS-associated biomarkers, suggesting their reduced CRS potential (Fig. 8J to M and Fig. S9A). This may be attributed to their innate-like immune features (Fig. S3) and the ability of NKT cells to recognize and eliminate CD1d^+^ macrophages involved in CRS pathogenesis [28,42,103,104]. In addition, we measured human CRS-related cytokines (i.e., IFN-γ, TNF-α, IL-2, GM-CSF, and IL-6) in mouse serum collected from the xenograft mouse model. All 3 therapeutic cell types demonstrated robust in vivo secretion of proinflammatory cytokines, including IFN-γ, TNF-α, and IL-2, supporting their active engagement with tumor cells and effective effector function (Fig. S9B). Notably, compared to ^PBMC15^MCAR-NKT cells and, particularly, conventional MCAR-T cells, ^Allo15^MCAR-NKT cells generated substantially lower levels of human IL-6 and GM-CSF, suggesting a reduced potential for CRS-associated toxicity while maintaining antitumor functionality (Fig. S9B) [104,109,110]. These findings further indicate a T helper 1 (Th1)-skewed profile for ^Allo15^MCAR-NKT cells (Fig. 2 and Fig. S2), consistent with their potent antitumor activity.

Neurotoxicity is an emerging concern for CAR-T cell therapies, especially due to their ability to breach the blood–brain barrier (BBB) and infiltrate the central nervous system (CNS) [98,110,111]. To evaluate brain infiltration, FG-labeled therapeutic cells were injected into NSG mice, and brain tissues were collected 30 d later. Tissue BLI revealed that only MCAR-T cells crossed the BBB and accumulated in the brain, while ^Allo15^MCAR-NKT and ^PBMC15^MCAR-NKT cells were excluded from the CNS (Fig. 8N to P). These data suggest a reduced risk of neurotoxicity for CAR-NKT cell therapies.

To comprehensively evaluate systemic toxicity, we measured serum levels of several organ damage biomarkers, including alanine aminotransferase (ALT) and aspartate aminotransferase (AST) as indicators of hepatocellular injury, total bilirubin as a marker of hepatic dysfunction or cholestasis, glutamate dehydrogenase (GLDH) as a liver-specific enzyme reflecting mitochondrial damage, and urea as a marker of renal function (Fig. S9C). Mice treated with conventional MCAR-T cells showed significantly elevated levels of all these markers, indicative of multi-organ toxicity, particularly affecting the liver and kidneys (Fig. S9C). In contrast, mice treated with ^Allo15^MCAR-NKT or ^PBMC15^MCAR-NKT cells maintained baseline levels of these biomarkers (Fig. S9C), supporting a low systemic toxicity profile and highlighting the high safety of these NKT cell-based therapies.

Next, we assessed the potential toxicity of ^Allo15^MCAR-NKT cells toward HSPCs and their impact on bone marrow recovery (Fig. S9D to L). First, we analyzed the bone marrow of experimental mice that had received ^Allo15^MCAR-NKT cells and evaluated the effects on mouse HSPC subsets, including long-term HSPCs (LT-HSPCs), short-term HSPCs (ST-HSPCs), and multi-potent progenitor cells (MPPs) (Fig. S9D). All 3 populations were preserved, indicating that ^Allo15^MCAR-NKT cells did not target these progenitors (Fig. S9E and F). Consistently, these murine HSPCs lacked CD1d expression, supporting the absence of direct recognition by ^Allo15^MCAR-NKT cells (Fig. S9G). Second, we confirmed this finding using human CD34^+^ HSPCs (Fig. S9H). Similar to the mouse cells, human LT-HSPCs, ST-HSPCs, and MPPs did not express CD1d (Fig. S9I and J). Coculture assays with ^Allo15^MCAR-NKT cells demonstrated no cytotoxicity against these human progenitors, further supporting the safety profile of this therapeutic approach (Fig. S9K and L). Nevertheless, we acknowledge that the mature myeloid cells express CD1d and could potentially be targeted by ^Allo15^MCAR-NKT cells. However, given the allogeneic nature of these therapeutic cells, which are expected to be rejected after the intended therapeutic window—as is the case with other allogeneic cell products—we do not anticipate a substantial long-term impact on HSPCs or bone marrow recovery [16,17].

To further improve the safety profile, we incorporated a suicide gene, sr39TK, into ^Allo^MCAR-NKT cells (denoted ^Allo^MCAR-NKT/TK cells) (Fig. S10A). sr39TK encodes a thymidine kinase that sensitizes transduced cells to ganciclovir (GCV)-induced apoptosis [112,113]. Upon GCV treatment, ^Allo^MCAR-NKT/TK cells underwent efficient elimination both in vitro and in vivo (Fig. S10B to E), validating the utility of this safety switch for rapid clinical control of cell therapy.

In summary, ^Allo15^MCAR-NKT cells demonstrate a favorable safety profile across multiple parameters, including a low incidence of xenogeneic GvHD and CRS, minimal neurotoxicity, limited off-target organ damage, and effective incorporation of a functional suicide switch. Collectively, these findings support the translational potential of ^Allo15^MCAR-NKT cells as a next-generation, off-the-shelf allogeneic CAR-NKT therapy for MPM and other solid malignancies.

### ^Allo15^MCAR-NKT cells exhibit hypoimmunogenic properties and resist host cell-mediated allorejection

Host-versus-graft responses represent a major barrier to the clinical application of allogeneic cell therapies [86,114]. In particular, host T cells and NK cells can recognize allogeneic therapeutic cells through mismatched human leukocyte antigen (HLA) molecules and stress-induced ligands, leading to immune activation, cytotoxicity, and eventual graft rejection. Therefore, minimizing immunogenicity while maintaining therapeutic function is a key objective in the development of “off-the-shelf” cellular therapies [115].

Building on our previous work demonstrating that HSPC-derived NKT cells exhibit reduced expression of HLA class I, HLA class II, and NK cell-activating ligands [28], we sought to determine whether ^Allo15^MCAR-NKT cells retain this hypoimmunogenic phenotype. We compared ^Allo15^MCAR-NKT cells with ^PBMC15^MCAR-NKT cells and conventional MCAR-T cells.

To evaluate host T cell-mediated alloreactivity, we performed in vitro MLR assays in which irradiated therapeutic cells were cocultured with HLA-mismatched PBMCs (Fig. S11A). IFN-γ production was used as a readout of allogeneic T cell activation. Across PBMCs from 8 independent donors, robust alloreactive responses were observed against ^PBMC15^MCAR-NKT cells and MCAR-T cells, whereas significantly reduced responses were detected against ^Allo15^MCAR-NKT cells, indicating diminished T cell-mediated recognition (Fig. S11B).

We next assessed susceptibility to NK cell-mediated rejection by coculturing freshly isolated PBMC-derived NK cells with the 3 therapeutic cell types (Fig. S11C). After 24 h, NK cell cytotoxicity was evaluated. While ^PBMC15^MCAR-NKT cells and MCAR-T cells were efficiently lysed by NK cells (with donor-dependent variability), ^Allo15^MCAR-NKT cells exhibited marked resistance to NK cell-mediated killing across all donors tested (Fig. S11D). These findings suggest that ^Allo15^MCAR-NKT cells evade both adaptive and innate immune surveillance mechanisms.

To investigate the underlying mechanisms, we analyzed the expression of immunologically relevant surface molecules, including HLA-I, HLA-II, and NK receptor ligands (e.g., MICA/B and ULBP family members). Prior to infusion, ^Allo15^MCAR-NKT cells expressed substantially lower levels of these molecules compared to ^PBMC15^MCAR-NKT and MCAR-T cells, as confirmed by both flow cytometry and scRNA-seq analyses (Fig. S11E to G). Notably, this low-expression profile was maintained in tumor-infiltrating ^Allo15^MCAR-NKT cells recovered in vivo, indicating phenotypic stability under physiological conditions (Fig. S11H).

Collectively, these results suggest that the reduced and stable expression of HLA molecules and NK-activating ligands confers a hypoimmunogenic phenotype to ^Allo15^MCAR-NKT cells, enabling them to resist host T cell- and NK cell-mediated allorejection and potentially supporting improved persistence in vivo (Fig. S11I). Importantly, residual expression of certain molecules, such as HLA-I, was still detectable, which may account for the low but measurable level of alloreactivity observed (Fig. S11E to H). This partial immunogenicity suggests that additional genetic engineering strategies, such as targeted disruption of β2-microglobulin (B2M) or CIITA to further reduce HLA expression, could be explored to further minimize host immune recognition and enhance persistence in future studies [33,107,116].

## Discussion

We describe the development of a scalable, allogeneic cell therapy platform based on human HSPCs, engineered to express an MSLN-directed CAR in NKT cells for the treatment of MPM. The resulting product demonstrates robust manufacturing scalability, high cellular purity, strong cytolytic function, and an encouraging safety profile. Leveraging a feeder-free HSPC modification and differentiation system, we reproducibly generated ^Allo15^MCAR-NKT cells with consistent phenotypic and functional characteristics across multiple CB-derived donors. These cells exhibited robust expansion kinetics, maintained transduction efficiency, and achieved >97% purity, meeting key benchmarks for clinical-grade, off-the-shelf cell therapy (Fig. 1J to M). Importantly, ^Allo15^MCAR-NKT cells demonstrated phenotypic stability and functional reproducibility across donor batches and manufacturing strategies (Fig. 1J to M). This reliability supports the broad utility of our platform for next-generation CAR engineering. In addition to MSLN, the platform can be readily adapted to target other mesothelioma-associated antigens such as EGFR, HER2, FAP, and MET (Fig. 1A to I) [117,118]. Furthermore, the lentiviral construct accommodates modular gene cassettes, enabling the incorporation of immunomodulatory payloads such as cytokines (e.g., IL-9, IL-10, IL-12, IL-18, and IL-21), chemokine receptors (e.g., CXCR3 and CCR5), or metabolic regulators (e.g., PGC-1α) to further optimize therapeutic fitness for the harsh mesothelioma TME [119–124]. Altogether, these features make ^Allo15^MCAR-NKT a versatile and clinically adaptable immunotherapy platform.

MPM presents unique immunotherapeutic challenges, including tumor antigen heterogeneity, poor immune infiltration, a myeloid-rich immunosuppressive TME, and substantial toxicity risk with conventional CAR-T cell strategies [125,126]. In this study, we compared ^Allo15^MCAR-NKT cells to healthy donor-derived conventional CAR-T cells and CAR-NKT cells across multiple functional assays. Our data show that ^Allo15^MCAR-NKT cells possess enhanced cytotoxic activity, greater tumor trafficking, lower exhaustion, and reduced immune checkpoint expression, which are all critical attributes for durable efficacy in TME-rich solid tumors such as MPM (Figs. 3, 5, and 6). Of note, we acknowledge that the current in vivo experiments were conducted using high doses of cell therapy products, including conventional MCAR-T cells. While ^Allo15^MCAR-NKT cells achieved effective tumor control, conventional MCAR-T cells ultimately induced xenogeneic GvHD, leading to mouse morbidity and death. Importantly, this xenogeneic GvHD may not be relevant in the clinic with autologous CAR-T cell therapy [106,127]. Additionally, evaluating lower and safer dosing regimens will be important to optimize therapeutic efficacy and minimize potential toxicity.

Antigen loss or heterogeneity is a well-recognized mechanism of resistance in mesothelioma and other solid tumors [71,87,94]. While MSLN is highly expressed in most MPM tumors, antigen density can vary, and down-regulation has been observed following therapeutic pressure. Unlike conventional CAR-T cells, ^Allo15^MCAR-NKT cells integrate multiple tumor recognition pathways, including CAR-mediated, NKR-mediated, and CD1d-restricted TCR-mediated killing (Fig. 3A to N). We demonstrated that ^Allo15^MCAR-NKT cells effectively lysed MSLN-low and MSLN-knockout tumor cells in vitro via NKG2D and DNAM-1 pathways and retained cytotoxic function against CD1d^+^ tumor cells through their invariant TCR (Fig. 3). This multimodal recognition mechanism positions ^Allo15^MCAR-NKT cells to overcome tumor heterogeneity and minimize immune evasion, a major limitation of monovalent conventional CAR-T cell therapies.

Tumor trafficking and infiltration remain key bottlenecks for CAR-T therapies in solid tumors, especially for MPM. The pleural tumor architecture, dense stroma, and immunosuppressive microenvironment can limit immune cell penetration and persistence [128,129]. To address this, a phase I trial tested intrapleural delivery of autologous MSLN-targeted CAR-T cells in patients with MPM for safety and local CAR-T accumulation [14]. However, in clinical setting, intrapleural delivery is limited by short-term CAR-T cell persistence, restricted access to extrapleural or metastatic sites, and the logistical challenges of repeated regional administration [14]. Our study showed that ^Allo15^MCAR-NKT cells exhibit robust tumor-homing behavior, accumulating preferentially in tumor tissues across subcutaneous and metastatic MPM models (Figs. 5F and 6F). This tumor-selective localization may be attributed to the unique tissue-homing profile of HSPC-derived CAR-NKT cells, which display high levels of chemokine receptors such as CXCR3 and CCR4, and a hybrid T and NK transcriptional program (Fig. 2) [28,42]. Importantly, this focused biodistribution was associated with potent antitumor activity and minimal off-target accumulation in liver or brain (Figs. 5F and 7P), supporting both efficacy and safety.

Moreover, we explored combination therapy with anti-TIM-3 checkpoint blockade, given the modest but consistent expression of TIM-3 on tumor-infiltrating ^Allo15^MCAR-NKT cells (Figs. 5G and 6G). This combination further improved tumor killing and experimental mice survival in a metastatic human MPM xenograft mouse model (Fig. 6), indicating that checkpoint inhibition can augment ^Allo15^MCAR-NKT cell activity even in the context of low baseline exhaustion. These data suggest a path forward for clinical development of rational combinatorial regimens. Indeed, sustained PD-1, LAG-3, TIM-3, and TIGIT signaling directly constrains NKT proliferation, cytokine secretion, and cytotoxicity, whereas lowering or blocking these checkpoints restores function [130–132]. For example, in a murine B16F10 melanoma metastasis model, NKT cell treatment with PD-1/PD-L1 blockade reduced the tumor nodule number compared with the control group [130]. Moreover, analyses of healthy donor PBMCs identified inverse relationships between LAG-3 or PD-1 expression and NKT proliferation, and both the anti-PD-1 monotherapy and dual anti-PD-1 and anti-LAG-3 increased NKT cell proliferation with elevated IFN-γ production, suggesting the potential of checkpoint blockade in NKT cell therapy [132–135].

Although our data demonstrate that TIM-3 blockade enhances ^Allo15^MCAR-NKT cell activity against H226-FG tumors expressing galectin-9 (Fig. 6), we recognize that the contribution of the TIM-3/galectin-9 axis may vary across tumor types [136,137]. In particular, the functional relevance of this pathway in galectin-9-low or galectin-9-negative tumors remains to be fully defined. Systematic evaluation using galectin-9 knockout or inhibitor-treated tumor models will be important for determining the breadth of responsiveness to this combination strategy. In addition, given that T cell exhaustion is a multidimensional and dynamic state governed by coordinated transcriptional, epigenetic, and functional programs rather than the expression of individual inhibitory receptors alone, we agree that low checkpoint expression is insufficient to define a “low exhaustion” phenotype in ^Allo15^MCAR-NKT cells. While our current data primarily focus on phenotypic and mechanistic correlates, future studies incorporating functional assays and exhaustion-associated transcriptional programs (e.g., TOX, NR4A family) will further delineate this relationship.

While our study demonstrates robust antitumor activity of ^Allo15^MCAR-NKT cells, it does not include direct comparisons with current standard-of-care therapies for MPM, such as chemotherapy in combination with immune checkpoint inhibitors (e.g., pembrolizumab + cisplatin/carboplatin + pemetrexed) [126,138]. Such comparisons would be valuable for more precisely defining the relative clinical positioning of this approach. However, evaluating these modalities in immunodeficient xenograft models presents inherent limitations, as their efficacy relies on intact and functional host immune systems that are not fully recapitulated in this setting. Future studies using more physiologically relevant models, including humanized or syngeneic systems, will be important to enable meaningful head-to-head comparisons and to better assess the translational potential of this off-the-shelf cellular therapy.

Despite clinical success in hematologic malignancies, CAR-T cell therapies face major safety challenges in solid tumors, including CRS, neurotoxicity, and GvHD for allogeneic settings [6,71,139,140]. These risks are especially concerning in MPM, where the inflamed pleural space increases vulnerability to systemic toxicity. Conventional MCAR-T cells often induce severe CRS and neurotoxicity, limiting their clinical utility. Encouragingly, our in vivo safety studies showed that ^Allo15^MCAR-NKT cells do not trigger GvHD, CRS, or neurotoxicity (Fig. 8 and Fig. S9). In contrast, conventional MCAR-T cells induced lethal GvHD and elevated proinflammatory cytokines in tumor-bearing mice (Fig. 8 and Fig. S9). The reduced toxicity of ^Allo15^MCAR-NKT cells stems from their inability to recognize mismatched MHCs via their semi-invariant TCR and their ability to eliminate CRS-promoting myeloid cells [42,56]. We also incorporated a suicide gene (i.e., sr39TK) into the CAR construct, enabling rapid clearance of the therapeutic cells with GCV if needed, further enhancing clinical safety (Fig. S10).

Lastly, our study demonstrates robust antitumor activity and favorable safety features of ^Allo15^MCAR-NKT cells in preclinical mouse models. Additional studies will be important to further strengthen clinical translation. In particular, evaluation in patient-derived xenograft (PDX) models may provide added insight into therapeutic efficacy in the context of preserved tumor heterogeneity. However, it is important to note that conventional PDX models in immunodeficient hosts lack key components of the human immune system, which may limit their ability to fully capture immune-mediated MOAs. Similarly, safety and pharmacokinetic assessment in nonhuman primate models could provide valuable information regarding in vivo persistence, biodistribution, and potential toxicity. Nonetheless, such studies are associated with inherent challenges, including species-specific differences in immune recognition and cytokine signaling, as well as potential incompatibility of target antigens, which may complicate data interpretation. Therefore, future investigations using advanced preclinical models, including humanized systems, as well as early-phase clinical studies, will be essential to more comprehensively evaluate the translational potential of this off-the-shelf cellular therapy.

In summary, ^Allo15^MCAR-NKT cells combine the benefits of scalable, off-the-shelf manufacturing, with multimodal tumor targeting, rejuvenated potential, and a strong safety profile. By addressing key barriers to solid tumor immunotherapy, such as antigen escape, poor infiltration, suppressive TME-induced exhaustion, and toxicity, our study lays the foundation for clinical translation of HSPC-engineered MCAR-NKT cell therapies in MPM. Future work will focus on optimizing dosing regimens, evaluating persistence in clinical models, and advancing toward first-in-human trials.

## Methods

Additional methods are included in the Supplementary Materials file.

### Mice

NOD.Cg-Prkdc^scid^Il2rg^tm1Wjl^/SzJ (NOD/SCID/IL-2Rγ^−/−^, NSG) (strain #005557, RRID: IMSR_JAX:005557), NOD.Cg-Prkdc^scid^ Il2rg^tm1Wjl^ Tg(CMV-IL3,CSF2,KITLG)1Eav/MloySzJ (NSG-SGM3) (strain #013062, RRID: IMSR_JAX:013062), and NOD.Cg-Prkdc^scid^ H2-K1^b-tm1Bpe^ H2-Ab1^g7-em1Mvw^ H2-D1^b-tm1Bpe^ Il2rg^tm1Wjl^/SzJ (NSG-MHC I/II DKO) (strain #025216, RRID: IMSR_ JAX:025216) mice were purchased from The Jackson Laboratory and maintained in University of California, Los Angeles (UCLA) animal facilities in a temperature-controlled environment (68 to 79 °F) with a 12-h light cycle. Six- to10-week-old mice were used for all experiments. In the human MPM xenograft mouse model, both male and female mice were used. No sex-related differences were observed in MPM tumor growth or therapeutic efficacy, and therefore, sex was not considered a variable in the experimental design. All mice were bred and maintained under specific pathogen-free conditions. All in vivo studies were conducted following approval from the UCLA Institutional Animal Care and Use Committee (IACUC). Experimental procedures involving animals adhered to the guidelines and regulatory standards established by the UCLA Division of Laboratory Animal Medicine (DLAM) (ARC protocol: ARC-2013-054). For all the in vivo studies, sample sizes were selected based on prior experience with similar tumor models and designed to provide sufficient statistical power to detect treatment effects. Mice were randomly assigned to experimental groups at the time of treatment to minimize allocation bias. Investigators conducting tumor measurements and survival monitoring were blinded to treatment groups. All mice inoculated with tumor cells and treated according to the study design were included in the analyses; no animals were excluded unless they met predefined humane endpoints unrelated to treatment efficacy.

### In vitro M2 macrophage-killing assay

M2-polarized macrophages were generated for use in the assay. Healthy donor PBMCs were suspended in serum-free RPMI 1640 at 1 × 10^7^ cells/ml, plated in 10-cm dishes (10 to 15 ml per dish), and incubated for 1 h at 37 °C with 5% CO₂. Non-adherent cells were discarded, and the remaining adherent monocytes were rinsed twice with phosphate-buffered saline (PBS). The cells were subsequently maintained in C10 medium supplemented with recombinant human macrophage colony-stimulating factor (M-CSF; 10 ng/ml; PeproTech, 300-25) for 6 d to differentiate into macrophages. On day 6, macrophages were detached using 0.25% trypsin/EDTA (Gibco, 25200-056), collected, and replated into 6-well plates (0.5 × 10^6^ to 1 × 10^6^ cells/ml) for 48 h with recombinant human IL-4 (10 ng/ml; PeproTech, catalog no. 214-14) and IL-13 (10 ng/ml; PeproTech, catalog no. 200-13) to induce M2 polarization. M2 macrophages were harvested for flow cytometry or for coculture assays.

For the in vitro macrophage-killing assay, M2 macrophages (1 × 10^5^ cells per well) were seeded into 96-well round-bottom plates and cocultured with therapeutic cells (i.e., ^Allo15^MCAR-NKT, ^PBMC15^MCAR-NKT, and MCAR-T cells; 1 × 10^5^ cells per well) at a 1:1 ratio in C10 medium for 24 h. Following coculture, cells were harvested and subjected to flow cytometric analysis to determine macrophage viability. To investigate NKT cell cytotoxicity mediated by the TCR/CD1d axis, αGC (100 ng/ml) and a blocking anti-human CD1d antibody (10 μg/ml; BioLegend, clone 51.1, catalog no. 350302, RRID: AB_10641848) were introduced into the coculture system. A corresponding isotype control antibody (mouse IgG2b κ; BioLegend, clone MG2b-57, catalog no. 401202, RRID: AB_2744505) was used as a negative control.

### In vitro MPM tumor organoid targeting assay

Healthy donor PBMC-derived, M2-polarized macrophages were used for this assay. Tumor organoids were generated by resuspending either 2 × 10^5^ H226 tumor cells alone or a 1:1 mixture of 1 × 10^5^ H226 tumor cells and 1 × 10^5^ M2 macrophages in C10 medium at 1 × 10^5^ cells/μl. Cell aggregates were formed by dispensing 5 to 10 μl of the suspension onto microporous membrane inserts (EMD Millipore, PICM0RG50) placed in 6-well plates containing 1 ml of C10 medium per well [95,96]. After a 2-d incubation to allow organoid formation, 1 × 10^6^ therapeutic cells (i.e., ^Allo15^MCAR-NKT, ^PBMC15^MCAR-NKT, and MCAR-T cells) in 100 μl of C10 medium were gently applied onto each organoid and cocultured for 24 h. After the coculture period, organoids were disrupted by repeated pipetting with a P1000 pipette, followed by passage through a 70-μm nylon mesh to generate single-cell suspensions for subsequent flow cytometric evaluation. It should be noted that this model does not represent classical patient-derived organoids generated with defined growth factor-driven differentiation. Rather, the structures formed in this assay are 3D tumor spheroid-like aggregates generated from established cell lines under non-adherent conditions. This simplified system was intentionally used to facilitate controlled tumor–macrophage and immune cell interactions while maintaining compatibility with downstream functional assays.

### In vivo evaluation of antitumor activity of ^Allo15^MCAR-NKT cells in an H226-FG human MPM subcutaneous xenograft NSG mouse model

An H226-FG human MPM subcutaneous xenograft model was established to evaluate the tumor homing and infiltration capacity of therapeutic cells. Briefly, 1 × 10^6^ H226-FG tumor cells expressing firefly luciferase and EGFP were subcutaneously injected into the right flank of NSG mice on day 0 to establish solid tumors. On day 4 post-tumor implantation, mice were randomized into treatment groups. Control animals received 100 μl of sterile 1× PBS via intravenous injection. Treatment groups received 1 × 10^7^ therapeutic cells, including ^Allo15^MCAR-NKT cells, ^PBMC15^MCAR-NKT cells, or conventional MCAR-T cells, administered intravenously. Where applicable, CAR-positive cells were either enriched by sorting or normalized prior to infusion to ensure comparable CAR^+^ cell dosing across groups. Tumor progression was monitored longitudinally by BLI and caliper-based measurements, and overall survival was recorded. In a parallel cohort, mice were euthanized on day 60 post-tumor implantation for tissue collection. Tumors and relevant organs were harvested, and infiltrating immune cell populations were characterized by flow cytometry.

In a separate experiment, 3 × 10^6^ H226-FG tumor cells were subcutaneously injected into the right flank of NSG mice on day 0 to establish solid tumors. A relatively higher tumor cell inoculum was used in this model to promote robust and consistent tumor growth. On day 4 post-implantation, mice were randomized into treatment groups. Control animals received 100 μl of sterile 1× PBS via subcutaneous injection. Treatment groups received therapeutic cells at 3 dose levels (1 × 10^6^, 3 × 10^6^, and 1 × 10^7^ cells), including ^Allo15^MCAR-NKT cells, ^PBMC15^MCAR-NKT cells, or conventional MCAR-T cells, administered via peritumoral injection. The use of multiple dose levels enabled evaluation of dose-dependent effects on antitumor efficacy and safety. Where applicable, CAR-positive cells were enriched by sorting or normalized prior to infusion to ensure equivalent CAR^+^ cell dosing across groups. Tumor progression was monitored longitudinally by caliper measurements. On day 35, mice were euthanized, and tumors were harvested for weight measurement.

All animal studies were conducted under IACUC-approved protocols with predefined humane endpoints, including clinical signs consistent with GvHD (e.g., weight loss >20%, hunching, ruffled fur, reduced activity). Mice that reached these criteria were humanely euthanized. In the MCAR-T cell-treated group, the majority of animals met humane endpoints consistent with GvHD.

### In vivo evaluation of antitumor activity of ^Allo15^MCAR-NKT cells in an H226-FG human MPM metastatic xenograft NSG mouse model

An H226-FG human MPM intravenous xenograft model was established to recapitulate lung dissemination and mimic advanced-stage metastatic disease progression. Briefly, 1 × 10^6^ H226-FG tumor cells were intravenously injected into NSG mice on day 0 to establish metastatic tumors. On day 4 post-tumor implantation, mice were randomized into treatment groups. Control animals received 100 μl of sterile 1× PBS via intravenous injection. Treatment groups received 1 × 10^7^ therapeutic cells, including ^Allo15^MCAR-NKT cells, ^PBMC15^MCAR-NKT cells, or conventional MCAR-T cells, administered intravenously. Where applicable, CAR-positive cells were either enriched by sorting or normalized prior to infusion to ensure comparable CAR^+^ cell dosing across groups. Mice were subsequently monitored for overall survival, and tumor burden was evaluated longitudinally using BLI. In a separate cohort, animals were euthanized on day 50 for tissue harvest, and infiltrating cell populations were analyzed by flow cytometry.

### In vivo evaluation of antitumor activity of ^Allo15^MCAR-NKT cells in an MSTO-FG human MPM metastatic xenograft NSG mouse model

An additional MSTO-FG human MPM intravenous xenograft model was established to incorporate multiple tumor cell lines, thereby capturing tumor heterogeneity and enabling broader evaluation of the applicability of the therapeutic cells across distinct tumor contexts. Briefly, 1 × 10^6^ MSTO-FG tumor cells were intravenously injected into NSG mice on day 0 to establish metastatic tumors. On day 4 post-tumor implantation, mice were randomized into treatment groups. Control animals received 100 μl of sterile 1× PBS via intravenous injection. Treatment groups received 1 × 10^7^ therapeutic cells, including ^Allo15^MCAR-NKT cells, ^PBMC15^MCAR-NKT cells, or conventional MCAR-T cells, administered intravenously. Where applicable, CAR-positive cells were either enriched by sorting or normalized prior to infusion to ensure comparable CAR^+^ cell dosing across groups. Throughout the study, mice were monitored for survival, and tumor progression was assessed longitudinally by BLI.

### In vivo evaluation of antitumor activity of ^Allo15^MCAR-NKT cells combined with anti-TIM-3 antibody in an H226-FG human MPM metastatic xenograft NSG mouse model

The H226-FG lung metastatic model was further utilized to evaluate the therapeutic efficacy of ^Allo15^MCAR-NKT cells in combination with an anti-TIM-3 antibody, with the aim of enhancing antitumor activity and overcoming immune inhibitory signaling within the TME. Briefly, 1 × 10^6^ H226-FG tumor cells were intravenously injected into NSG mice on day 0 to establish metastatic tumors. On day 4 post-tumor implantation, mice were randomized into treatment groups. Control animals received 100 μl of sterile 1× PBS via intravenous injection. Treatment groups received 3 × 10^6 Allo15^MCAR-NKT cells, administered intravenously. In addition, mice were treated with an anti-TIM-3 antibody (sabatolimab biosimilar; Bio X Cell, catalog no. SIM0043, RRID: AB_3696382) administered intraperitoneally at 10 mg/kg on a weekly schedule. Throughout the study, survival was monitored, and tumor burden was evaluated longitudinally using BLI.

### In vivo evaluation of antitumor activity of ^Allo15^MCAR-NKT cells in a human ovarian cancer xenograft NSG mouse model

An OVCAR8-FG^MSLN−/−^ human ovarian cancer xenograft model lacking MSLN expression was employed to evaluate CAR-independent, NKR-mediated tumor cell killing by therapeutic cells. Intraperitoneal injection of ovarian cancer tumor cells was used to recapitulate key features of human ovarian cancer, including peritoneal dissemination, ascites formation, and tumor growth within the peritoneal cavity. Briefly, 3 × 10^6^ OVCAR8-FG^MSLN−/−^ tumor cells were intraperitoneally injected into NSG mice on day 0 to establish peritoneal tumors. On day 5 post-tumor implantation, mice were randomized into treatment groups. Control animals received 100 μl of sterile 1× PBS via intraperitoneal injection. Treatment groups received 1 × 10^7^ therapeutic cells, including ^Allo15^MCAR-NKT cells or PBMC-derived NK cells, administered intraperitoneally. Throughout the study, mice were monitored for survival, and tumor burden was assessed twice weekly using BLI.

### In vivo MPM TME targeting study of ^Allo15^MCAR-NKT cells: H226-FG human MPM metastatic xenograft NSG-SGM3 mouse model

NSG-SGM3 mice were utilized to establish a solid tumor model with enhanced TME representation and to evaluate TME-mediated modulation of therapeutic cell function. The NSG-SGM3 model expresses human cytokines, including SCF, GM-CSF, and IL-3, which support the development and maintenance of human myeloid and immune cell compartments, thereby enabling improved modeling of human TME interactions. Briefly, 1 × 10^6^ H226-FG tumor cells were intravenously injected into NSG-SGM3 mice on day 0 to establish the lung metastatic tumors. On day 4 post-tumor implantation, mice were randomized into treatment groups. Control animals received 100 μl of sterile 1× PBS via intravenous injection. Treatment groups received 1 × 10^7^ therapeutic cells, including ^Allo15^MCAR-NKT cells, ^PBMC15^MCAR-NKT cells, or conventional MCAR-T cells, administered intravenously. Where applicable, CAR-positive cells were either enriched by sorting or normalized prior to infusion to ensure comparable CAR^+^ cell dosing across groups. On days 3, 6, and 9, the experimental mice received intravenous injection of healthy donor PBMC-derived CD14^+^ myeloid cells (10 × 10^6^ cells in 100 μl of PBS per mouse). Note that these myeloid cells were derived from the same donor and purified by magnetic-activated cell sorting (MACS) using human CD14 MicroBeads (Miltenyi Biotech, catalog no. 130-097-052) to remove nonmyeloid populations. On day 12, experimental mice were euthanized, lungs were harvested, and isolated cells were analyzed by flow cytometry.

### In vivo GvHD evaluation study of ^Allo15^MCAR-NKT cells

Both standard NSG and NSG-MHC I/II DKO mice were employed to evaluate xenogeneic GvHD induced by therapeutic cells, enabling assessment across distinct levels of MHC-dependent immune interactions and providing a more comprehensive characterization of GvHD potential. On day 0, mice received 1 × 10^7^ therapeutic cells, including ^Allo15^MCAR-NKT cells, ^PBMC15^MCAR-NKT cells, or conventional MCAR-T cells, administered intravenously. Where applicable, CAR-positive cells were either enriched by sorting or normalized prior to infusion to ensure comparable CAR^+^ cell dosing across groups. Animals were subsequently monitored for survival, body weight changes, and the development of xenogeneic GvHD. Clinical GvHD severity was evaluated using a scoring system (0 to 2 per parameter) based on body weight, activity level, posture, skin thickening, diarrhea, and fur condition [84]. At study completion, multiple organs were harvested and processed for histopathological assessment.

### In vivo CRS evaluation study of ^Allo15^MCAR-NKT cells

On day 0, NSG mice were intraperitoneally implanted with H226-FG cells (5 × 10^6^ cells per mouse). Ten days later, animals received intraperitoneal administration of vehicle control (100 μl of PBS), ^Allo15^MCAR-NKT cells, ^PBMC15^MCAR-NKT cells, or MCAR-T cells (each at 1 × 10^7^ CAR^+^ cells in 100 μl of PBS per mouse). On day 13, blood and peritoneal lavage samples were collected for downstream analyses. Levels of IL-6 and SAA-3 in both serum and peritoneal fluid were quantified by ELISA. SAA-3 concentrations were measured using a commercially available Mouse SAA-3 ELISA kit (MilliporeSigma) according to the manufacturer’s protocol.

CRS-associated toxicity was also evaluated using a standardized clinical scoring system based on observable physiological and behavioral parameters. Severe endpoints (score 4) included ≥20% body weight loss, body condition score ≤2/5, severe dehydration, lack of response to external stimuli, inability to ambulate, lateral recumbency, and labored breathing. Moderate signs (score 2) included hypothermia. Mild clinical signs (score 1) included lethargy, hunched posture, ruffled fur, eye squinting, ascites, and mild to moderate dehydration persisting beyond 24 h despite fluid support. Any additional unlisted abnormal signs were evaluated in consultation with veterinary staff. This composite scoring system enabled quantitative assessment of CRS severity and humane endpoint determination.

### In vivo brain damage evaluation study of ^Allo15^MCAR-NKT cells

On day 0, the experimental mice received intraperitoneal injection of vehicle (100 μl of PBS per mouse), FG-labeled ^Allo15^MCAR-NKT/FG cells (10 × 10^6^ CAR^+^ cells in 100 μl of PBS per mouse), FG-labeled ^PBMC15^MCAR-NKT/FG cells (10 × 10^6^ CAR^+^ cells in 100 μl of PBS per mouse), or FG-labeled MCAR-T/FG cells (10 × 10^6^ CAR^+^ cells in 100 μl of PBS per mouse). On day 30, experimental mice were intraperitoneally injected with d-luciferin (10 mg per mouse), followed by euthanasia and collection of brain tissues for BLI.

### In vivo organ damage evaluation study of ^Allo15^MCAR-NKT cells

On day 0, NSG mice received 1 × 10^7^ therapeutic cells, including ^Allo15^MCAR-NKT cells, ^PBMC15^MCAR-NKT cells, or conventional MCAR-T cells, administered intravenously. Where applicable, CAR-positive cells were either enriched by sorting or normalized prior to infusion to ensure comparable CAR^+^ cell dosing across groups. Blood samples were collected from experimental mice on days 1, 20, and 40 for the assessment of organ injury biomarkers by ELISA. Blood urea nitrogen (BUN) levels were determined using a colorimetric assay kit (Thermo Fisher Scientific, catalog no. EIABUN). AST and ALT concentrations were measured using ELISA kits from Abcam (catalog nos. ab263882 and ab282882, respectively). Total bilirubin and glutamate dehydrogenase (GLDH) levels were quantified using ELISA kits obtained from MyBioSource (catalog nos. MBS3805359 and MBS761948, respectively).

To further evaluate the organ damage, experimental mice were euthanized and tissues were harvested for organ safety evaluation by hematoxylin and eosin (H&E) staining. Formalin-fixed, paraffin-embedded sections of bone marrow, spleen, ileum, pancreas, kidney, lung, liver, heart, and brain were examined for preservation of normal tissue architecture according to established organ-specific morphological features. Inflammation was evaluated by identifying granulocytic infiltration, typically localized around blood vessels due to lymphocyte extravasation, and scored based on the number and severity of inflammatory foci: 0 (0 to 2 sites of minor inflammation), 1 (3 to 10 sites of minor inflammation or 1 to 2 sites of moderate inflammation), 2 (10 to 20 sites of minor inflammation or 3 to 5 sites of moderate inflammation), and 3 (≥20 sites of minor inflammation or extensive, diffuse inflammation). Neoplastic changes were assessed using predefined histopathological criteria, including enlarged nuclei with increased nuclear-to-cytoplasmic ratio, irregular nuclear morphology or chromatin structure, increased mitotic figures or prominent nucleoli, loss of cell polarity, disorganized cellular clustering, loss of cellular heterogeneity, and presence of tumor-associated vasculature. Neoplasm scores were assigned according to the total number of criteria observed: 0 (none), 1 (one criterion), 2 (2 to 3 criteria), and 3 (4 or more criteria). When present, neoplasms were further classified as hematopoietic (HN), characterized by small, darkly stained immature or dedifferentiated lymphoid or granulocytic cells, or nonhematopoietic (NHN), defined as atypical proliferations of tissue-specific parenchymal cells. Each organ was scored independently for inflammation, HN, and NHN, with higher scores indicating greater tissue injury severity.

## Ethical Approval

This study complies with all relevant ethical regulations. Animal studies were approved by the Division of Laboratory Animal Medicine at UCLA (ARC-2013-054). Healthy donor PBMCs were provided by the UCLA/CFAR Virology Core Laboratory without identification information under federal and state regulations.

## Data Availability

All data needed to evaluate the conclusions in the paper are present in the paper and/or the Supplementary Materials. Reagent requests should be directed to L.Y. (liliyang@ucla.edu) and copied to Y.-R.L. (charlie.li@ucla.edu). The scRNA-seq data generated or reanalyzed in this study (related to Fig. 2 and Figs. 2A to C and Fig. S2 and S3) have been deposited in the public repository Gene Expression Omnibus Database (GSE305915). The Methyl-seq data generated or reanalyzed in this study have been deposited in the public repository Gene Expression Omnibus Database (GSE251909 and GSE306014). The scRNA-seq publicly available data used in this study (related to Fig. 1) are available in the public repository Gene Expression Omnibus Database (GSE190597).
