# Supplementary material for: Rejuvenated Hematopoietic Stem and Progenitor Cell-Engineered CAR-Armored Natural Killer T Cells for Malignant Pleural Mesothelioma
Source: Research (Wash D C). 2026 Jun 11;9:1310. doi: 10.34133/research.1310 (PMC13254501; doi:10.34133/research.1310)
Supplement: Supplementary 1 — Additional Methods Figs. S1 to S11 Table S1 [file research.1310.f1.docx]

**Supplementary Materials**

**Rejuvenated Hematopoietic Stem and Progenitor Cell-Engineered CAR-Armored Natural Killer T Cells for Malignant Pleural Mesothelioma**

Yan-Ruide Li, Yichen Zhu, Zhe Li, Xinyuan Shen, Shuo Li, Yuning Chen, Zibai Lyu, Jie Huang, Nathan Y. Ma, Catherine Zhang, Annabel S. Zhao, Yanxin Tian, Xianghong Jasmine Zhou, Lili Yang

**Additional methods**

**Media and reagents**

The X-VIVO 15 Serum-Free Hematopoietic Cell Medium was purchased from Lonza. The StemSpan T cell Generation Kit, including the StemSpan SFEM II Medium, the StemSpan Lymphoid Progenitor Expansion Supplement, the StemSpan Lymphoid Progenitor Maturation Supplement, the StemSpan Lymphoid Progenitor Differentiation Coating Material, and the ImmunoCult Human CD3/CD28/CD2 T Cell Activator, was purchased from STEMCELL Technologies. The CTS OpTmizer T Cell Expansion SFM (no phenol red, bottle format) was purchased from Thermo Fisher Scientific. αGC (KRN7000) was purchased from Avanti Polar Lipids. Recombinant human IL-2, IL-3, IL-7, IL-15, IL-21, IFN-γ, Flt3 ligand (Flt3L), stem cell factor (SCF), and thrombopoietin (TPO) were purchased from PeproTech. Ganciclover (GCV), fetal bovine serum (FBS), and β-mercaptoethanol (β-ME) were purchased from Sigma. Penicillin-streptomycin-glutamine (P/S/G), MEM nonessential amino acids (NEAA), HEPES buffer solution, and sodium pyruvate were purchased from Gibco. Normocin was purchased from InvivoGen. The RPMI 1640 cell culture medium and the DMEM cell culture medium were purchased from Thermo Fisher Scientific. The CryoStor Cell Cryopreservation Media CS10 was purchased from MilliporeSigma.

The C10 medium was made of RPMI 1640 cell culture medium supplemented with FBS (10% v/v), P/S/G (1% v/v), NEAA (1% v/v), HEPES (10 mM), sodium pyruvate (1 mM),β-ME (50 mM), and Normocin (100 mg/mL). The C10 medium was used to culture human T and NKT cells. The D10 medium was made of DMEM supplemented with FBS (10% v/v), P/S/G (1% v/v), and Normocin (100 mg/mL). The D10 medium was used to culture human 293T cells. The R10 medium was made of RPMI 1640 supplemented with FBS (10% v/v), P/S/G (1% v/v), and Normocin (100 mg/mL). The R10 medium was used to culture other human tumor cells.

**Cell lines**

Human MM cell line MM.1S (cat. no. CRL-2974, RRID: CVCL_8792), chronic myelogenous leukemia cell line K562 (cat. no. CCL-243, RRID: CVCL_0004), melanoma cell line A375 (cat. no. CRL-1619, RRID: CVCL_0132), mesothelioma cell line H226 (cat. no. CRL-5826, RRID: CVCL_1544), mesothelioma cell line MSTO-211H (MSTO; cat. no. CRL-2081, RRID: CVCL_1430), human embryonic kidney cell line HEK293T (cat. no. CRL-3216, RRID: CVCL_0063) were purchased from the ATCC. Ovarian cancer cell lines OVCAR8 was obtained from the NIH. The parental tumor cell lines were transduced with lentiviral vectors encoding the intended gene(s) to produce stable tumor cell lines overexpressing firefly luciferase and enhanced green fluorescent protein dual reporters (FG), human MSLN, or human CD1d. 72 hours post lentivector transduction, cells were subjected to flow cytometry sorting to isolate gene-engineered cells for generating stable cell lines. Ten stable tumor cell lines were generated for this study, including MM-FG, MM-MSLN-FG, H226-FG, MSTO-FG, OVCAR8-FG, A375-FG, K562-FG, H226-FG^MSLN-/-^, OVCAR8-FG^MSLN-/-^, and H226-CD1d-FG^MSLN-/-^ cell lines. The H226-FG^MSLN-/-^ and OVCAR8-FG^MSLN-/-^ cell lines were generated by knocking out the *MSLN* gene from the parental H226-FG and OVCAR8 cell lines using CRISPR-Cas9. The single guide RNA targeting the *MSLN* gene (UAUUAAGC UCAGUCCCAAAC) was purchased from Synthego, and was introduced into parental tumor cells via electroporation using an Amaxa 4D Nucleofection X Unit (Lonza), according to the manufacturer’s instructions. The H226-CD1d-FG^MSLN-/-^ cell line was further transduced with Lenti/CD1d vector to overexpress human CD1d.

The artificial antigen-presenting cell line (aAPC) was generated by engineering the K562 human chronic myelogenous leukemia cell line to overexpress human CD80/CD83/CD86/4-1BBL co-stimulatory receptors [1]. The aAPC-MSLN cell lines were generated by further engineering the parental aAPC line to overexpress human MSLN.

**Lentiviral vectors**

All lentiviral vectors used in this study were constructed from a parental vector pMNDW. The 2A sequences derived from foot-and-mouth disease virus (F2A), porcine teschovirus-1 (P2A), and thosea asigna virus (T2A) were used to link the inserted genes to achieve co-expression.

Six lentivectors were constructed and used in this study [2]. The Lenti/iNKT-MCAR-IL-15 vector was constructed by inserting into the pMNDW vector a synthetic tetracistronic gene encoding human iNKT TCRα-F2A-TCRβ-P2A-MCAR-T2A-IL-15 (MCAR indicates a mesothelin-targeting CAR, and IL-15 indicates the secreting form of human IL-15). The Lenti/iNKT-MCAR-sr39TK vector was constructed by inserting into the pMNDW vector a synthetic tetracistronic gene encoding human iNKT TCRα-F2A-TCRβ-P2A-MCAR-T2A sr39TK (sr39TK indicates an sr39TK suicide and positron emission tomography imaging reporter gene) [3]. The Lenti/MCAR vector was constructed by inserting a synthetic gene encoding MCAR into the pMNDW. The Lenti/FG vector was constructed by inserting a synthetic bicistronic gene encoding Fluc-P2A-EGFP into the pMNDW. The Lenti/CD1d vector was constructed by inserting a synthetic gene encoding human CD1d into the pMNDW. The Lenti/MSLN vector was constructed by inserting a synthetic gene encoding mesothelin into the pMNDW. The Lenti/STAT1C-EGFP vector was constructed by inserting into the pMNDW parental vector a synthetic bicistronic gene encoding human STAT1C-P2A-EGFP (STAT1C indicates a human constitutively active STAT1) [4]. The synthetic gene fragments were obtained from GenScript and IDT. Lentiviruses were produced using human embryonic kidney 293T (HEK293T) cells (ATCC), following a standard transfection protocol using the Trans-IT-Lenti Transfection Reagent (Mirus Bio) and a centrifugation concentration protocol using the Amicon Ultra Centrifugal Filter Units, according to the manufacturer’s instructions (MilliporeSigma)

**Antibodies and flow cytometry**

Fluorochrome-conjugated antibodies specific for human CD3 (clone HIT3a, Pacific Blue, APC, PE, or PE-Cy7-conjugated, 1:500, cat. no. 300330, 300311, 300308, or 300316, RRID: AB_10551436, AB_314047, AB_314044, or AB_314052), CD4 (clone OKT4, PE-Cy7, PerCP, APC, or FITC-conjugated, 1:500, cat. no. 317414, 317432, 317415, or 317408, RRID: AB_571959, AB_2028494, AB_571944, or AB_571951), CD5 (clone L17F12, APC, Pacific Blue, or FITC-conjugated, 1:500, cat. no. 364015, 364024, or 364021, RRID: AB_2565725, AB_2566250, or AB_2566247), CD7 (clone CD7-6B7, FITC or APC-conjugated, 1:500, cat. no. 343103 or 343107, RRID: AB_1659217 or AB_1877156), CD8 (clone SK1, PE or APC-conjugated, 1:300, cat. no. 980902 or 344721, RRID: AB_ 2616623 or AB_2075390), CD28 (clone CD28.2, APC, FITC, or Pacific Blue-conjugated, 1:200, cat. no. 302911, 302906, or 302928, RRID: AB_ 314313, AB_314308, or AB_10641279), CD45 (clone HI30, PerCP, FITC or Pacific Blue-conjugated, 1:500, cat. no. 304025, 304005, or 304021, RRID: AB_893341, AB_314393, or AB_493654), CD56 (clone QA18A21, APC-Cy7 or PE-conjugated, 1:20, cat. no. 398813 or 398803, RRID: AB_3097438 or AB_2820070), CD69 (clone FN50, PE-Cy7 or PerCP-conjugated, 1:50, cat. no. 310911 or 310927, RRID: AB_ 314846 or AB_10696423), CD112 (clone TX31, APC or PE-conjugated, 1:200, cat. no. 337411 or 337409, RRID: AB_2565729 or AB_2174163), CD155 (clone SKII.4, PE-Cy7-conjugated, 1:250, cat. no. 337613, RRID: AB_2565746), CD1d (clone 51.1, PE-Cy7 or APC-conjugated,1:50, cat. no. 25-0016-42 or 17-0016042, RRID: AB_2815094 or AB_11219079), TCRαβ (clone IP26, Pacific Blue or PE-Cy7-conjugated, 1:25, cat. no. 306715 or 306719, RRID: AB_1953256 or AB_10640829), MICA/MICB (clone 6D4, APC-conjugated, 1:25, cat. no. 320907, RRID: AB_493196), IFN-γ (clone B27, PE-Cy7-conjugated, 1:50, cat. no. 506517, RRID: AB_2123322), NKG2D (clone 1D11, PE-Cy7-conjugated, 1:50, cat. no. 320811, RRID: AB_2133275), DNAM-1 (clone 11A8, APC-conjugated, 1:50, cat. no. 338311, RRID: AB_2561951), NKp30 (clone P30-15, APC-conjugated, 1:50, cat. no. 325209, RRID: AB_2149450), NKp44 (clone P44-8, PE-Cy7-conjugated, 1:50, cat. no. 325115, RRID: AB_2616753), KIR (clone HP-MA4, FITC, APC, or PE-conjugated, 1:50, cat. no. 339503, 339509, or 339505, RRID: AB_1501076, AB_ 2565576, or AB_ 2130376), Granzyme B (clone QA16A02, APC-conjugated, 1:2,000 or 1:5,000, cat. no. 372203, RRID: AB_2687027), Perforin (clone dG9, PE-Cy7-conjugated, 1:50 or 1:100, cat. no. 308125, RRID: AB_ 2572048), IL-2 (clone MQ1-17H12, APC-Cy7-conjugated, 1:50, cat. no. 500341, RRID: AB_ 2562854), PD-1 (clone A17188A, FITC or PE-conjugated, 1:50, cat. no. 379205 or 379209, RRID: AB_2922605 or AB_2922607), LAG-3 (clone 11C3C65, FITC, APC-Cy7, or PE-conjugated, 1:50, cat. no. 369307, 369347, or 369305, RRID: AB_2629750, AB_2922585, or AB_2629591), TIM-3 (clone A18087E, PE or APC-conjugated, 1:50, cat. no. 364805 or 364803, RRID: AB_2922577 or AB_2910409), TIGIT (clone A15153G, PE, APC, or APC-Cy7-conjugated, 1:50, cat. no. 372703, 372705, or 372733, RRID: AB_2632729, AB_2632731, or AB_2876700), and CTLA-4 (clone BNI3, PE-Cy7 or APC-cy7-conjugated, 1:50, cat. no. 369613 or 369633, RRID: AB_2632875 or AB_2892451) were purchased from BioLegend. Fluorochrome-conjugated antibodies specific for mouse c-kit (clone ACK2, APC-Cy7-conjugated, 1:50, cat. no. 135135, RRID: AB_2632808), Sca1 (clone D7, APC-conjugated, 1:50, cat. no. 108112, RRID: AB_313349), Flt3 (clone A2F10, PE-conjugated, 1:50, cat. no. 135306, RRID: AB_1073482), SLAM (clone TC15-12F12.2, PE-Cy7-conjugated, 1:50, cat. no. 115914, RRID: AB_439797), and CD1d (clone 1B1, FITC-conjugated, 1:50, cat. no. 123508, RRID: AB_1236549) were purchased from BioLegend. Fluorochrome-conjugated antibodies specific for human CD34 (clone 581, PE-conjugated, 1:500, cat. no. 555822, RRID: AB_396151) was purchased from BD Biosciences. Fluorochrome-conjugated antibody specific for human iNKT TCR Vβ11 (clone C21, APC-conjugated, 1:50, cat. no. A66905, RRID: AB_3683578) was purchased from Beckman Coulter. Fluorochrome-conjugated antibodies specific for human ULBP-1 (clone 170818, PE-conjugated, 1:25, cat. no. FAB1380P, RRID: AB_2687471), ULBP-2,5,6 (clone 165903, APC-conjugated, 1:25, cat. no. FAB1298A, RRID: 2257142), and mesothelin (clone 420411, PE-conjugated, 1:10, cat. no. FAB32652P, RRID: 1151946) were purchased from R&D Systems. A goat anti-mouse IgG F(ab’)2 secondary antibody (HRP-conjugated, 1:50, cat. no. 31436, RRID: 228313) was purchased from Thermo Fisher Scientific. Fixable Viability Dye eFluor506 (e506, 1:500, cat. no. 65-0866-18) was purchased from Affymetrix eBioscience. Mouse Fc Block (anti-mouse CD16/32, clone 2.4G2, 1:50, cat. no. 553141, RRID: 394656) was purchased from BD Biosciences, and human Fc Receptor Blocking Solution (TrueStain FcX, cat. no. 422302, RRID: 2818986) was purchased from BioLegend. In our study, note the use of antibodies with identical clones but differing conjugated fluorochromes, with one typical antibody listed in the table. In addition, as the same markers were assessed under multiple experimental conditions (e.g., *in vitro* and *in vivo*), antibody staining protocols were optimized for each condition to ensure signal comparability. Accordingly, antibody dilutions were adjusted as needed based on the specific experimental context. For simplicity, only a representative dilution is listed above.

All flow cytometry staining was performed following standard protocols, as well as specific instructions provided by the manufacturer of a particular antibody. Stained cells were analyzed using a MACSQuant Analyzer 10 flow cytometer (Miltenyi Biotech), following the manufacturers’ instructions. FlowJo software version 9 (BD Biosciences) was used for data analysis. For intracellular cytokine staining, the cells were thawed and resuspended in C10 medium. Cells were stimulated with PMA (Calbiochem, cat. no. 524400; 50 ng/mL) and ionomycin (Calbiochem, cat. no. 407952.; 500 ng/mL) and incubated at 37°C for 2 hours. GolgiStop (BD Biosciences, car. No. 554724; 1.5 µL/mL) was then added to inhibit cytokine secretion, followed by an additional 4-hour incubation. Subsequently, intracellular staining was performed using the Cell Fixation/Permeabilization Kit (BD Biosciences, cat. no. 554714) according to the manufacturer’s instructions.

**Human cord blood CD34^+^ hematopoietic stem and progenitor Cells (HSPCs) and periphery blood mononuclear cells (PBMCs)**

Purified CB-derived human CD34^+^ HSPCs were purchased from HemaCare. Healthy donor PBMCs were provided by the UCLA/CFAR Virology Core Laboratory without identification information under federal and state regulations.

**Enzyme-linked immunosorbent cytokine assays (ELISAs)**

The ELISAs for detecting human cytokines were performed following a standard protocol from BD Biosciences. Supernatants from cell culture assays were collected and assayed to quantify human IFN-γ, TNF-α, and IL-2. The capture and biotinylated pairs for detecting cytokines were purchased from BD Biosciences. The streptavidin-HRP conjugate was purchased from Invitrogen. Human cytokine standards were purchased from eBioscience. Tetramethylbenzidine substrate was purchased from KPL. Human IL-15 was quantified using a Human IL-15 Quantikine ELISA Kit (R&D Systems), following the manufacturer’s instructions. Mouse IL-6 was quantified with paired purified anti-mouse IL-6 antibody and biotin anti-mouse IL-6 antibody (BioLegend). Mouse SAA-3 was quantified using a Mouse SAA-3 ELISA Kit (MilliporeSigma), as per the manufacturer’s instructions. The samples were analyzed for absorbance at 450 nm using an Infinite M1000 microplate reader (Tecan).

**Generation of HSPC-derived allogeneic IL-15-enhanced MCAR-engineered NKT (^Allo15^MCAR-NKT) cells**

^Allo15^MCAR-NKT cells were generated from gene-engineered human cord blood CD34^+^ HSPCs using a 5-stage clinically guided culture method [1,4]. The starting HSPCs were engineered to overexpress a transgenic human iNKT TCR, a MSLN-targeting CAR and the secreted form of human IL-15. The detailed protocols have been previously described [1,2]; here, we summarize the key steps involved in generating ^Allo15^MCAR-NKT cells

At Stage 0, frozen-thawed human CD34^+^ HSPCs were revived and cultured in X-VIVO 15 Serum-Free Hematopoietic Stem Cell Medium supplemented with Flt3L (50 ng/ml), SCF (50 ng/ml), TPO (50 ng/ml), and IL-3 (20 ng/ml) in non-tissue culture treated 24-well plate for 24 hours, then transduced with Lenti/iNKT-MCAR-IL-15 viruses for another 24 hours following a previously established protocol [1,3,5]. To generate FG-engineered ^Allo15^MCAR-NKT/FG cells, HSPCs were co-transduced with Lenti/iNKT-MCAR-IL-15 and Lenti/FG viruses. To generate sr39TK-engineered ^Allo^MCAR-NKT/TK cells, HSPCs were transduced with Lenti/iNKT-MCAR-sr39TK viruses. Concentrated lentiviral supernatants were supplemented with Poloxamer Synperonic F108 (Sigma-Aldrich, cat. no. 07579-250G-F) and Prostaglandin E2 (PGE2; Cayman Chemical, cat. no. 140110) to enhance transduction efficiency, and subsequently added to the HSPC cultures with gentle mixing.

At Stage 1, gene-engineered HSPCs collected from Stage 0 were cultured in the StemSpan^TM^ SFEM II Medium supplemented with StemSpan^TM^ Lymphoid Progenitor Expansion Supplement (denoted as LPE medium) for two weeks. CELLSTAR®24-well Cell Culture Nontreated Multiwell Plates (VWR) were used. The plates were pre-coated with 500 µl/well StemSpan^TM^ Lymphoid Differentiation Coating Material for 2 hours at room temperature or alternatively, overnight at 4 °C. Gene-engineered HSPCs were suspended at 2 x 10^4^ cells/ml and 500 µl of cell suspension was added into each pre-coated well. Twice per week, half of the medium from each well was removed and replaced with fresh LPE medium.

At Stage 2, cells collected from the Stage 1 were cultured in the StemSpan^TM^ SFEM II Medium supplemented with StemSpan^TM^ Lymphoid Progenitor Maturation Supplement (denoted as LPM medium) for one week. Non-Treated Falcon™ Polystyrene 6-well Microplates (ThermoFisher Scientific) were coated with 1 ml/well of StemSpan^TM^ Lymphoid Differentiation Coating Material. The Stage 1 cells were collected and resuspended at 1 x 10^5^ cells/ml; 2 ml of cell suspension was added into each pre-coated well. Cells were passaged 1-2 times per week to maintain a cell density at 0.5-1 x 10^6^ cells/ml; fresh LPM medium was added at every passage.

At Stage 3, the cells collected from the Stage 2 were cultured in the LPM medium supplemented with CD3/CD28/CD2 T Cell Activator and 20 ng/ml human recombinant IL-15 for one week. Cells were resuspended at 5 x 10^5^ cells/ml; 2 ml cell suspension was added into Non-Treated Falcon™ Polystyrene 6-well Microplates pre-coated with 1 ml/well of StemSpan^TM^ Lymphoid Differentiation Coating Material. Cells were passaged 2-3 times per week to maintain a cell density at 0.5-1 x 10^6^ cells/ml; fresh LPM medium supplemented with human IL-15 was added at every passage.

At Stage 4, cells collected from the Stage 3, now mature ^Allo/15^MCAR-NKT cells, were expanded using the following three approaches: (a) an αCD3/αCD28 antibody-based expansion, (b) an αGC/PBMC-based expansion, or (c) an artificial APC (aAPC)-based expansion [1,2]. The ^Allo15^MCAR-NKT cells used in most assays of this study were expanded using the aAPC-based expansion method. aAPCs were irradiated at 10,000 rads using a Rad Source RS-2000 X-Ray Irradiator (Rad Source Technologies). Mature ^Allo15^MCAR-NKT cells collected from the Stage 3 culture were mixed with the irradiated aAPCs at 1:1 - 1:2 ratio, resuspended in expansion medium supplemented with 10 ng/ml IL-7 and IL-15 at 0.5-1 x 10^6^ cells/ml, and seeded into the 150-mm cell culture dishes (ThermoFisher Scientific) at 30 ml per plate. Cells were passaged 2-3 times per week to maintain a cell density at 0.5-1 x 10^6^ cells/ml; fresh expansion medium was added at every passage. The expansion stage lasted for 2 weeks. At stage 4, cells could be cultured in 150-mm cell culture dishes (ThermoFisher Scientific) or G-Rex^®^6M Well Plates (Wilson Wolf). The expansion can happen in a feeder-free, serum-free CTS™ OpTmizer™ T-Cell Expansion SFM (ThermoFisher Scientific), or a homemade C10 medium. The resulting ^Allo15^MCAR-NKT cell products were aliquoted and cryopreserved in CryoStor® Cell Cryopreservation Media CS10 using a Thermo Scientific™ CryoMed™ Controlled-Rate Freezer 7450 (Thermo scientific) for future use, following the manufacturers' instructions.

In this study, all three expansion approaches were used to generate ^Allo15^MCAR-NKT cells. However, all functional assays, including *in vitro* and *in vivo* tumor killing studies, were performed using ^Allo15^MCAR-NKT cells produced with the aAPC-based expansion protocol.

**Generation of PBMC-derived conventional αβ T, NKT, and NK cells**

Healthy donor PBMCs were used to generate the PBMC-derived conventional αβ T, NKT, and NK cells (denoted as PBMC-T cells, PBMC-NKT cells, and PBMC-NK cells, respectively) [4].

To generate PBMC-T cells, PBMCs were stimulated with the Dynabeads^TM^ Human T-Activator CD3/CD28 (ThermoFisher Scientific) according to the manufacturer’s instructions, followed by culturing in the C10 medium supplemented with human IL-2 (20 ng/mL) for 2–3 weeks.

To generate PBMC-NKT cells, PBMCs were MACS-sorted via Anti-iNKT MicroBeads (Miltenyi Biotech) labeling to enrich NKT cells, following the manufacturer’s instructions. The enriched NKT cells were mixed with donor-matched irradiated αGC-loaded PBMCs at a ratio of 1:1 - 1:2, followed by culturing in C10 medium supplemented with 10 ng/ml IL-7 and IL-15 for 2-3 weeks. If needed, the resulting cultured cells could be further purified using Fluorescence-Activated Cell Sorting (FACS) via human NKT TCR antibody (clone 6B11; BD Biosciences) staining.

To generate PBMC-NK cells, PBMCs were FACS-sorted using a FACSAria™ III Sorter (BD Biosciences) via human CD56 antibody (Clone HCD56; BioLegend, cat. no. 343758, RRID: AB_3683360) labeling, or MACS-sorted using a Human NK Cell Isolation Kit (Miltenyi Biotech), following the manufacturers’ instructions.

**Generation of MCAR-engineered conventional αβ T (MCAR-T) cells**

Non-treated tissue culture 24-well or 12-well plates (Corning) were coated with Ultra-LEAF™ Purified Anti-Human CD3 Antibody (Clone OKT3; BioLegend, cat. no. 317302, RRID: AB_571927) at 1 µg/ml (500 µl/well), at room temperature for 2 hours or alternatively, overnight at 4 °C. Healthy donor PBMCs were resuspended in the C10 medium supplemented with 1 µg/ml Ultra-LEAF™ Purified Anti-Human CD28 Antibody (Clone CD28.2, BioLegend, cat. no. 377203, RRID: AB_3097476) and 30 ng/ml IL-2, followed by seeding in the pre-coated plates at 1 x 10^6^ cells/ml (1 ml/well). On day 2, cells were transduced with Lenti/MCAR virus for 24 hours. The resulting MCAR-T cells were expanded for about 2 weeks in C10 medium supplemented with human IL-2, and cryopreserved for future use, following established protocols [1,4,5].

In this study, CAR⁺ cells were pre-sorted for all *in vivo* antitumor efficacy experiments and for scRNA-seq analyses. For the *in vitro* tumor cell killing assays, the number of conventional MCAR-T cells was adjusted by normalizing to the CAR⁺ cell fraction, given that non–CAR-transduced T cells do not exhibit cytotoxic activity in short-term assays. For all flow cytometry analyses, CAR⁺ cells were pre-gated, and their phenotype and functional properties were subsequently evaluated.

**Generation of PBMC-derived IL-15-enhanced MCAR-engineered NKT (^PBMC15^MCAR-NKT) cells**

Healthy donor PBMCs were sorted with MACS via Anti-iNKT Microbeads (Miltenyi Biotech) labeling to enrich NKT cells, following the manufacturer’s instructions. The enriched NKT cells were mixed with donor-matched irradiated αGC/PBMCs at a ratio of 1:1 - 1:2, followed by culturing in C10 medium supplemented with 10 ng/ml IL-7 and IL-15. On day 3, NKT cells were transduced with Lenti/MCAR-IL-15 viruses for 24 h. The resulting CAR-NKT cells were expanded for about 2 weeks in C10 medium supplemented with 10 ng/ml IL-7 and IL-15 and cryopreserved for future use. In this study, ^PBMC15^MCAR-NKT and conventional MCAR-T cells were generated from multiple (>8) healthy donor PBMCs, including some unmatched donors.

**Generation of PBMC-derived IL-15-enhanced MCAR-engineered NK (^PBMC15^MCAR-NK) cells**

Healthy donor PBMCs were sorted with MACS via a Human NK Cell Isolation Kit (Miltenyi Biotech) to enrich NK cells, following the manufacturer’s instructions. The enriched NK cells were mixed with irradiated aAPCs at a ratio of 1:10, followed by culturing in C10 medium supplemented with 10 ng/ml IL-7 and IL-15. On day 3, NK cells were transduced with Lenti/MCAR-IL15 viruses for 24 h. The resulting ^PBMC15^MCAR-NK cells were expanded for about 1 week in C10 medium supplemented with 10 ng/ml IL-7 and IL-15.

**Methylation sequencing (Methyl-seq)**

9 samples including 3 ^Allo15^MCAR-NKT, 3 ^PBMC15^MCAR-NKT, and 3 MCAR-T cell samples were analyzed using Methyl-seq. Genomic DNA was isolated from experimental samples using a QIAGEN DNeasy Blood & Tissue kit, then sonicated using a Covaris M220 Focused-ultrasonicator, following the manufacturers’ instructions. DNA fragments of around 250 bp were enriched using the Ampure XP beads (Beckman-Coulter), then subjected to DNA library preparation using an NEBNext Ultra II DNA library prep Kit (Cat#E7645) following the manufacturers’ instructions. The DNA libraries were then subjected to sequencing on Illumina NovaSeq sequencer with 2 x 150 bp configuration (Azenta). The raw bisulfite sequencing reads were trimmed by cutadapt to remove sequencing adapters. The trimmed reads were then aligned to hg19 reference genome by Bismark [6]. Then duplicated reads from PCR amplification were identified and removed by Bismark. The deduplicated reads were then sorted and indexed using Samtools [7]. After that, the methylated and unmethylated cytosines were counted at every CpG site by Bismark. The methylation at a gene promoter region was quantified as the beta value, i.e., the ratio between the number of methylated cytosines and the total number of cytosines mapped to the region. The promoter region of a gene was identified by GeneHancer with the highest confidence score. The beta values were then used for heatmap visualization.

**Single cell RNA sequencing (scRNA-seq)**

Four therapeutic cell samples (i.e., ^Allo15^MCAR-NKT, ^PBMC15^MCAR-NKT, MCAR-T, and PBMC-NK cells) were analyzed by scRNA-seq. These cells, prepared as described above as the final cell products, were delivered to the UCLA TCGB Core for library construction and sequencing. These cells were derived from unmatched PBMC donors, and such donor-to-donor variability may influence transcriptional profiles and introduce heterogeneity in functional assays. Nonetheless, additional experiments, including flow cytometry, *in vitro*, and *in vivo* assays, were performed using cells from multiple independent donors, and the consistent trends observed across these datasets support the robustness of our conclusions. These cells were then quantified using a Cell Countess II automated cell counter (Invitrogen/Thermo Fisher Scientific). A total of 10,000 cells from each experimental group were loaded on the Chromium platform (10X Genomics), and libraries were constructed using the Chromium Next GEM Single Cell 3' Kit v3.1 and the Chromium Next GEM Chip G Single Cell Kit (10X Genomics), according to the manufacturer’s instructions. Library quality was assessed using the D1000 ScreenTape on a 4200 TapeStation System (Agilent Technologies). Libraries were sequenced on an Illumina NovaSeq using the NovaSeq S4 Reagent Kit (100 cycles; Illumina). After quality control, a total of 14,747 ^Allo15^MCAR-NKT cells, 4,667 ^PBMC15^MCAR-NKT cells, 12,595 MCAR-T cells, and 1,577 PBMC-NK cells were included in the analysis.

In another study, scRNA-seq was utilized to examine the gene profiles of primary MPM patient-derived malignant cells. Data from Gene Expression Omnibus database (GSE190597) were included for scRNA-seq analyses [8]. A total of 30,101 cells were included in the analysis. Cell clustering was performed according to previously studies [8,9].

For cell clustering and annotation, the merged digital expression matrix generated by Cellranger was analyzed using an R package Seurat (v.4.0.0) following the official website guidelines [10–12]. Briefly, after filtering the low-quality cells, the expression matrix was normalized using NormalizeData function, followed by selecting top 2,000 most variable genes across datasets using FindVariableFeatures and SelectIntegrationFeatures functions. To correct the batch effect, FindIntegrrationAnchors and IntegrateData functions were used based on the selected feature genes. The corrected dataset was subjected to standard Seurat workflow for dimension reduction and clustering. In this study, clusters of therapeutic cells were manually merged and annotated based on gene signatures reported from Human Protein Atlas (proteinatlas.org) and previous studies related to T and NKT cells [13–20]. AddModuleScore was used to calculate module scores of each list of gene signatures, and FeaturePlot function was used to visualize the expression of each signature in the UMAP plots. For gene set enrichment analysis (GSEA), clusterProfiler packages [21,22] were used to calculate the enrichment scores of each cluster in the signature gene list.

**Western blot**

Western blot was used to analyze the immune checkpoint expression and IFN-γ signaling events in ^Allo15^MCAR-NKT cells. ^PBMC15^MCAR-NKT, and conventional MCAR-T cells. To analyze immune checkpoint expression, therapeutic cells were isolated via MACS sorting following the *in vitro* repeated tumor challenge assay and subsequently subjected to Western blot analysis. To analyze IFN-γ signaling events, the cells were stimulated with IFN-γ (10 ng/ml) for 15 minutes (for blotting p-STAT1/STAT1), or 18 hours (for blotting IRF-1), and then collected.

Total proteins were extracted using a RIPA lysis buffer (Thermo Fisher Scientific) containing 20 mM HEPES (pH 7.6), 150 mM NaCl, 1mM EDTA, 1% Tritonx-100, and protease/phosphatase inhibitor cocktail (Cell Signaling Technology). Protein concentration was measured using a Bicinchoninic Acid (BCA) Assay Kit (Thermo Fisher Scientific). Equal amounts of total protein were resolved on a 4–15% Mini-PROTEAN® TGX™ Precast Protein Gel (BIO-RAD) and then transferred to a polyvinylidene difluoride (PVDF) membrane by electrophoresis. The following antibodies were used to blot for the proteins of interest: anti-human PD-1 (clone D4W2J, Cell Signaling Technology, CST, cat. no. 86163S, RRID: AB_2728833), anti-human CTLA-4 (clone E2V1Z, CST, cat. no. 53560S, RRID: AB_3107072), anti-human TIM-3 (clone D5D5R, CST, cat. no. 45208S, RRID: AB_2716862), anti-human LAG-3 (clone D2G4O, CST, cat. no. 15372S, RRID: AB_2798739), anti-human TIGIT (clone E5Y1W, CST, cat. no. 99567S, RRID: AB_2922806), anti-human p-STAT1(Y701) (clone 58D6, CST, cat. no. 9167S, RRID: AB_561284), anti-human STAT1 (clone D1K9Y, CST, cat. no. 14994S, RRID: AB_2737027), anti-human IRF-1 (clone D5E4, CST, cat. no. 8478S, RRID: AB_10949108), and secondary anti-rabbit IgG (CST, cat. no. 7074S, RRID: AB_2099233). β-Actin (clone D6A8, CST, cat. no. 8457S, RRID: AB_10950489) was used as internal controls. Signals were visualized using a ChemiDoc^™^ Imaging Systems (BIO-RAD). The data were analyzed using ImageJ (Version 1.53s).

***In vitro* tumor cell killing assay**

Tumor cells (1 $\times$ 10^4^ cells per well) were co-cultured with effector cells (at ratios indicated in the figure legends) in Corning 96-well clear bottom black plates for 24 h, in C10 medium with or without the addition of αGC (100 ng/mL). D-luciferin (150 mg/mL, Caliper Life Science) was added to cell cultures to quantify live tumor cells and luciferase activities were read out using an Infinite M1000 microplate reader (Tecan). In tumor killing assays involving blocking CD1d, 10 mg/mL LEAF purified anti-human CD1d antibody (clone 51.1, BioLegend, cat. no. 350322, RRID: AB_2814281) or LEAF purified mouse lgG2bk isotype control antibody (clone MG2B-57, BioLegend, cat. no. 401202, RRID: AB_2744505) was added to tumor cell cultures 1 h prior to adding ^Allo15^MCAR-NKT cells. In experiments that study the NKR-mediated tumor cell killing mechanism, 10 mg/mL LEAF purified anti-human NKG2D (clone 1D11, BioLegend, cat. no. 320814, RRID: AB_2561488), anti-human DNAM-1 antibody (clone 11A8, BioLegend, cat. no. 639754, RRID: AB_3662324), or LEAF purified mouse lgG2bk isotype control antibody was added to co-cultures.

***In vitro* serial tumor cell killing assay**

A total of 1 $\times$ 10^4^ non-FG-engineered tumor cells (e.g., H226 cells; referred to as stimulator cells) was co-cultured with 2 $\times$ 10^5^ effector cells in a Corning 96-well clear bottom black plate in C10 medium. Cultures were supplemented with a dose of 1 $\times$ 10^4^ stimulator cells every 2 days. 24 h prior to luminescent tumor killing readout, stimulator cells were substituted with 1 $\times$ 10^4^ of FG-engineered tumor cells (e.g., H226-FG cells; referred to as indicator cells). To quantify the remaining live indicator cells, 100 ml of D-luciferin (10 mg/ml) was added to cell cultures on the day of imaging and the luciferase activities were measured through readout with an Infinite M1000 microplate reader (Tecan).

***In vitro* MLR assay: Studying GvH response**

PBMCs from more than ten random healthy donors were irradiated at 2,500 rads and used as stimulators to investigate the GvH response of ^Allo15^MCAR-NKT and ^PBMC15^MCAR-NKT cells as responders. PBMC-derived MCAR-T cells were included as a responder control. Stimulators (5 $\times$ 10^5^ cells/well) and responders (2 $\times$ 10^4^ cells/well) were co-cultured in 96-well round-bottom plates in C10 medium for 4 days; the cell culture supernatants were then collected to measure IFN-γ production using ELISA. Note that the IFN-γ production was solely attributed to ^Allo/PBMC15^MCAR-NKT or MCAR-T responder cells.

***In vivo* bioluminescence imaging (BLI)**

BLI was performed using a Spectral Advanced Molecular Imaging HTX system (Spectral Instrument Imaging). Live animal images were captured 5 minutes after intraperitoneal (i.p.) injection of D-Luciferin (1 mg per mouse) to obtain total body bioluminescence. Tissue images were captured 5 minutes after i.p. injection of D-luciferin (10 mg per mouse), followed by euthanasia, tissue collection, and imaging [23]. The imaging data were analyzed using AURA imaging software (version 3.2.0, Spectral Instrument Imaging).

***In vivo* GCV depletion assay**

The experimental design is shown in Figure S10C. On day 0, NSG mice received i.v. injection of ^Allo^MCAR-NKT/TK cells, followed by i.p. injection of GCV for 5 consecutive days (50 mg/kg per injection per day). On day 5, mice were terminated. Multiple tissues (i.e., blood, spleen, liver, and lung) were collected and processed for flow cytometry analysis to detect circulating and tissue-infiltrating ^Allo^MCAR-NKT/TK cells (identified as NKT TCR^+^CD45^+^CD3^+^ cells).

**Histology analysis**

Tissues (i.e., livers and lungs) were harvested from experimental mice, fixed in 10% neutral buffered formalin for up to 36 h, and embedded in paraffin for sectioning (5 mm thickness). Tissue sections were subsequently prepared and stained with hematoxylin and eosin, anti-GFP (Abcam, cat. no. ab183734, RRID: AB_2732027), or anti-human CD3 (DAKO, cat. no. A0452, RRID: AB_2335677) by the UCLA Translational Pathology Core Laboratory (TPCL) in accordance with the Core’s standard protocols. Stained sections were imaged using an Olympus BX51 upright microscope equipped with an Optronics Macrofire CCD camera (AU Optronics), and the images were analyzed using an Optronics PictureFrame software (AU Optronics).

**Statistics**

Statistical data analysis was performed using GraphPad Prism 8 software (GraphPad). Student’s two-tailed *t* test was employed for pairwise comparisons. Ordinary one- or two-way ANOVA followed by Tukey’s or Dunnett’s multiple comparisons test was used for multiple comparisons. Log rank (Mantel-Cox) test adjusted for multiple comparisons was used for Meier survival curves analysis. Data are expressed as the mean ±SEM, unless otherwise indicated. In all figures and figure leg ends, n denotes the number of samples or animals utilized in the indicated experiments. A p-value of less than 0.05 was considered significant; ns indicates not significant; *p < 0.05, **p < 0.01, ***p < 0.001, ****p < 0.0001.

**References**

1. Li Y-R, Zhou Y, Yu J, Kim YJ, Li M, Lee D, et al. Generation of allogeneic CAR-NKT cells from hematopoietic stem and progenitor cells using a clinically guided culture method. Nat Biotechnol [Internet]. 2024; Available from: https://doi.org/10.1038/s41587-024-02226-y

2. Li Y-R, Zhou K, Lee D, Zhu Y, Halladay T, Yu J, et al. Generating allogeneic CAR-NKT cells for off-the-shelf cancer immunotherapy with genetically engineered HSP cells and feeder-free differentiation culture. Nat Protoc [Internet]. 2025; Available from: https://doi.org/10.1038/s41596-024-01077-w

3. Zhu Y, Smith DJ, Zhou Y, Li YR, Yu J, Lee D, et al. Development of Hematopoietic Stem Cell-Engineered Invariant Natural Killer T Cell Therapy for Cancer. Cell Stem Cell [Internet]. 2019;25:542-557.e9. Available from: https://doi.org/10.1016/j.stem.2019.08.004

4. Li Y-R, Li Z, Zhu Y, Li M, Chen Y, Lee D, et al. Overcoming ovarian cancer resistance and evasion to CAR-T cell therapy by harnessing allogeneic CAR-NKT cells. Med (New York, NY). 2025;100804.

5. Li Y-R, Zhou Y, Kim YJ, Zhu Y, Ma F, Yu J, et al. Development of allogeneic HSC-engineered iNKT cells for off-the-shelf cancer immunotherapy. Cell reports Med. 2021;2:100449.

6. Krueger F, Andrews SR. Bismark: a flexible aligner and methylation caller for Bisulfite-Seq applications. Bioinformatics. 2011;27:1571–2.

7. Li H, Handsaker B, Wysoker A, Fennell T, Ruan J, Homer N, et al. The Sequence Alignment/Map format and SAMtools. Bioinformatics. 2009;25:2078–9.

8. Giotti B, Dolasia K, Zhao W, Cai P, Sweeney R, Merritt E, et al. Single-Cell View of Tumor Microenvironment Gradients in Pleural Mesothelioma. Cancer Discov. 2024;14:2262–78.

9. Severson DT, Freyaldenhoven S, Wadowski B, Hung YP, Hughes T, Yeap BY, et al. Multi-omics, histologic, and scRNA-seq profiling of pleural mesothelioma reveals negative prognosis associated with a novel uncommitted molecular phenotype. J Thorac Oncol Off Publ Int Assoc Study Lung Cancer. 2025;

10. Butler A, Hoffman P, Smibert P, Papalexi E, Satija R. Integrating single-cell transcriptomic data across different conditions, technologies, and species. Nat Biotechnol [Internet]. 2018;36:411–20. Available from: https://doi.org/10.1038/nbt.4096

11. Tran HTN, Ang KS, Chevrier M, Zhang X, Lee NYS, Goh M, et al. A benchmark of batch-effect correction methods for single-cell RNA sequencing data. Genome Biol. 2020;21:12.

12. Jia C, Hu Y, Kelly D, Kim J, Li M, Zhang NR. Accounting for technical noise in differential expression analysis of single-cell RNA sequencing data. Nucleic Acids Res. 2017;45:10978–88.

13. Miller BC, Sen DR, Al Abosy R, Bi K, Virkud Y V, LaFleur MW, et al. Subsets of exhausted CD8(+) T cells differentially mediate tumor control and respond to checkpoint blockade. Nat Immunol. 2019;20:326–36.

14. Zheng L, Qin S, Si W, Wang A, Xing B, Gao R, et al. Pan-cancer single-cell landscape of tumor-infiltrating T cells. Science. 2021;374:abe6474.

15. Li H, van der Leun AM, Yofe I, Lubling Y, Gelbard-Solodkin D, van Akkooi ACJ, et al. Dysfunctional CD8 T Cells Form a Proliferative, Dynamically Regulated Compartment within Human Melanoma. Cell. 2019;176:775-789.e18.

16. Milner JJ, Toma C, He Z, Kurd NS, Nguyen QP, McDonald B, et al. Heterogenous Populations of Tissue-Resident CD8(+) T Cells Are Generated in Response to Infection and Malignancy. Immunity. 2020;52:808-824.e7.

17. Beltra J-C, Manne S, Abdel-Hakeem MS, Kurachi M, Giles JR, Chen Z, et al. Developmental Relationships of Four Exhausted CD8(+) T Cell Subsets Reveals Underlying Transcriptional and Epigenetic Landscape Control Mechanisms. Immunity. 2020;52:825-841.e8.

18. Zheng C, Zheng L, Yoo J-K, Guo H, Zhang Y, Guo X, et al. Landscape of Infiltrating T Cells in Liver Cancer Revealed by Single-Cell Sequencing. Cell. 2017;169:1342-1356.e16.

19. Wang X, Peticone C, Kotsopoulou E, Göttgens B, Calero-Nieto FJ. Single-cell transcriptome analysis of CAR T-cell products reveals subpopulations, stimulation, and exhaustion signatures. Oncoimmunology. 2021;10:1866287.

20. Bai Z, Woodhouse S, Zhao Z, Arya R, Govek K, Kim D, et al. Single-cell antigen-specific landscape of CAR T infusion product identifies determinants of CD19-positive relapse in patients with ALL. Sci Adv. 2022;8:eabj2820.

21. Yu G, Wang L-G, Han Y, He Q-Y. clusterProfiler: an R package for comparing biological themes among gene clusters. OMICS. 2012;16:284–7.

22. Wu T, Hu E, Xu S, Chen M, Guo P, Dai Z, et al. clusterProfiler 4.0: A universal enrichment tool for interpreting omics data. Innov (Cambridge. 2021;2:100141.

23. Lyu Z, Li Y-R, Yang L. Protocol for assessing pharmacokinetics and pharmacodynamics of human CAR-NKT cells in humanized mouse models using bioluminescence imaging. STAR Protoc. 2025;6:103957.





**Fig. S1. Generation of ^Allo15^MCAR-NKT cells; related to Fig. 1.**

(A) Table showing the stages and materials of the *Ex Vivo* HSPC-Derived CAR-NKT Cell Culture. Cryopreserved human cord blood-derived CD34^+^ hematopoietic stem and progenitor cells (HSPCs) were used to generate CAR-NKT cell products through a 5-stage, 6-week process. At Stage 0, HSPCs were transduced with a lentivector, and then cultured for 2 days in a X-VIVO 15-based serum-free HSPC Medium. Gene-engineered HSPCs were then cultured over ~6 weeks to generate the CAR-NKT cell product: Stage 1 HSPC expansion (~2 weeks), Stage 2 CAR-NKT differentiation (~1 week), Stage 3 CAR-NKT deep differentiation (~1 week), and Stage 4 CAR-NKT expansion (~2 weeks). The Stage 1 Culture Medium comprised the StemSpan^TM^ SFEM II Medium (SFEM) and the StemSpan^TM^ Lymphoid Progenitor Expansion Supplement to support the HSPC expansion. The Stage 2 Culture Medium comprised the SFEM and the StemSpan^TM^ Lymphoid Progenitor Maturation Supplement (LPMS) to support the NKT cell differentiation. The Stage 3 Culture Medium comprised the SFEM, the LPMS, the CD3/CD28/CD2 T Cell Activator, and the human recombinant IL-15 to support CAR-NKT cell deep differentiation. In addition, the StemSpan^TM^ Lymphoid Differentiation Coating Material were utilized throughout Stages 1 to 3 to support HSPC expansion and differentiation into T cell lineage. The entire 5-stage culture can be implemented in a feeder-free and serum-free manner. Alternatively, two feeder-dependent strategies involving α-galactosylceramide (αGC)-loaded healthy donor PBMCs or K562-based artificial antigen presenting cells (aAPCs) can be employed for Stage 4 CAR-NKT expansion.

(B and C) Kinetics and yields of ^Allo15^MCAR-NKT cells throughout the entire culture stages. (B) Table showing estimated input and output cell numbers for ^Allo15^MCAR-NKT cells. (C) Yields and fold changes of ^Allo15^MCAR-NKT cells over time. Data generated from 6 CB donors was shown.

(D-F) Comparison of different methods to expand ^Allo15^MCAR-NKT cells at Stage 4 (week 4 to week 6). (D) Expansion folds of ^Allo15^MCAR-NKT cells generated by three methods (n = 5; n indicates different CB donors). (E) FACS detection of the purity and CD4/CD8 coreceptor expression of ^Allo15^MCAR-NKT cells. (F) Expansion folds of ^Allo15^MCAR-NKT cells generated using aAPC method with or without serum (n = 3; n indicates different CB donors).

(G and H) Studying the IL-15 production by ^Allo15^MCAR-NKT cells. (G) Experimental design. ^Allo15^MCAR-NKT cells were stimulated with αGC-loaded healthy donor PBMCs and cultured for 48 hours, after which the culture supernatants were collected for ELISA analysis. (H) ELISA analyses of IL-15 production by ^Allo15^MCAR-NKT cells (n = 4).

(I and J) Studying the dysregulated growth of ^Allo15^MCAR-NKT cells. (I) Experimental design. Mature ^Allo15^MCAR-NKT cells were cultured with IL-7/IL-15, IL-7 alone, or no cytokines, and their cell numbers were counted. (J) ^Allo15^MCAR-NKT cell numbers during the 6-day culture (n = 4).

Representative of > 10 experiments. Data are presented as the mean ± SEM. ns, not significant; *p < 0.05; ****p < 0.0001, by Student’s *t* test (F and H), one-way ANOVA (D), or two-way ANOVA (J).

**

**

**Fig. S2. Generation and characterization of PBMC-derived CAR-engineered immune cells utilized in the study; related to Fig. 1.**

(A-B) Diagram showing the generation of PBMC-derived MCAR-engineered conventional αβ T (MCAR-T) cells (A), and PBMC-derived IL15-enhanced MCAR-engineered NKT (^PBMC15^MCAR-NKT) cells (B).

(C) Heatmap showing the all differentially expressed genes (DEGs) among the three therapeutic cell types.

(D) FACS analyses of the expression of NKRs on the indicated therapeutic cells (n = 5; n indicates different CB or PBMC donors).

(E) FACS analyses of the production of Th1 cytokines (i.e., IFN-γ, IL-2, and TNF-α), Th2 cytokine (i.e., IL-4) and cytotoxic molecules (i.e., Perforin and Granzyme B) by the indicated therapeutic cells (n = 5; n indicates different CB or PBMC donors).

Representative of 3 experiments. Data are presented as the mean ± SEM. ns, not significant; *p < 0.05; **p < 0.01; ***p < 0.001; ****p < 0.0001 by one-way ANOVA (D and E).

**Fig. S3. ^Allo15^MCAR-NKT cells display intrinsic and potent NK functions; related to Fig. 2.**

(A) Diagram showing the generation of PBMC-derived natural killer (PBMC-NK) cells.

(B-D) scRNA-seq comparison between ^Allo15^MCAR-NKT and PBMC-NK cells. (B) Violin plots showing the expression distribution of T and NK cell gene signatures in the indicated cells. p = 3 x 10^-7^ for T cell gene signature, and p < 2.2 x 10^-16^ for NK cell gene signature. (C) Violin plots showing the expression distribution of proliferation gene signature, memory gene signature, and exhausted gene signature in the indicated cells. p < 2.2 x 10^-16^ for all the gene signatures. (D) Violin plots showing the expression distribution of genes encoding NK/NKT transcription factor, activation marker, NK markers, proliferating markers, memory markers, and exhaustion markers in the indicated cells. p < 2.2 x 10^-16^ for all the genes.

(E and F) Comparison of the phenotype and functionality of ^Allo15^MCAR-NKT and PBMC-NK cells. (E) FACS detection of NK activating receptors, NK inhibitory receptors, and cytotoxic molecules in the indicated cells. (F) Quantification of (D) (n = 4; n indicates different CB or PBMC donors).

(G and H) Comparison of the *in vitro* antitumor efficacy of ^Allo15^MCAR-NKT and PBMC-NK cells. (G) Experimental design. (H) Tumor cell killing data at 24 h (n = 4).

(I-L) Comparison of the *in vivo* antitumor efficacy of ^Allo15^MCAR-NKT and PBMC-NK cells. (I) Experimental design. BLI, live animal bioluminescence imaging. (J) BLI images measuring tumor loads in experimental mice over time. (K) Quantification of (J) (n = 5). (L) Kaplan–Meier survival curves of experimental mice over time (n = 5).

Representative of 1 (B-D) and 3 (A, and E-L) experiments. Data are presented as the mean ± SEM. **p < 0.01; ***p < 0.001; ****p < 0.0001 by Student’s *t*-test (F), two-way ANOVA (H), one-way ANOVA (K), or log rank (Mantel-Cox) test adjusted for multiple comparisons (L). p values of violin plots were determined by Wilcoxon rank sum test (B-D).

**

**

**Fig. S4. Studying the *in vitro* tumor cell killing efficacy of ^Allo15^MCAR-NKT cells; related to Fig. 2 and 3.**

(A-F) Comparison between ^Allo15^MCAR-NKT and human PBMC-derived IL-15-enhanced MCAR-engineered NK (^PBMC15^MCAR-NK) cells.

(A) Schematics showing the generation of ^PBMC15^MCAR-NK cells.

(B) FACS plots showing the MCAR expression on ^PBMC15^MCAR-NK cells. Data from three independent PBMC donors are presented.

(C and D) Comparison of the *in vitro* antitumor efficacy of ^Allo15^MCAR-NKT and ^PBMC15^MCAR-NK cells using a 24-hour *in vitro* tumor cell killing assay. (C) Experimental design. (D) Tumor cell killing data at 24 h (n = 4).

(E-H) Comparison of the *in vitro* antitumor efficacy of ^Allo15^MCAR-NKT and ^PBMC15^MCAR-NK cells using a long-term *in vitro* serial tumor cell killing assay. (E) Experimental design. (F) Tumor cell killing data (n = 4). (G) Cell numbers of ^Allo15^MCAR-NKT and ^PBMC15^MCAR-NK cells (n = 4). (H) ELISA analyses of IFN-γ and TNF-α production by ^Allo15^MCAR-NKT and ^PBMC15^MCAR-NK cells in culture supernatants collected on day 21 of the co-culture assay (n = 4).

(I and J) Studying the tumor killing mechanism of ^Allo15^MCAR-NKT cells mediated by NKRs. (I) Tumor cell killing data by ^Allo15^MCAR-NKT cells at 24 h (n = 4). MSTO-FG, E:T ratio = 0.5:1; H226-FG^MLSN-/-^, E:T ratio = 2:1. (J) Tumor cell killing data by ^PBMC15^MCAR-NKT cells at 24 h (n = 4). H226-FG^MLSN-/-^, E:T ratio = 10:1.

Representative of 3 experiments. Data are presented as the mean ± SEM. ns, not significant; *p < 0.05; **p < 0.01; ***p < 0.001; ****p < 0.0001 by Student’s *t*-test (D and H), one-way ANOVA (I and J), or two-way ANOVA (F and G).

**

**

**Fig. S5. Studying the *in vivo* antitumor efficacy of ^Allo15^MCAR-NKT cells in an H226-FG subcutaneous human MPM xenograft NSG mouse model; related to Fig. 5.**

(A) Kaplan–Meier survival curves were generated from combined data of two independent experiments (Vehicle, n = 10; all other groups, n = 8). Survival endpoints were defined according to IACUC-approved humane criteria, including: (1) spontaneous death, (2) development of GvHD-associated signs such as >20% body-weight loss, hunching, ruffled fur, or reduced activity, prompting humane euthanasia, or (3) tumor burden exceeding 400 mm^3^ or BLI signal >2 × 10^10^, resulting in impaired mobility. Mice meeting any of these criteria were recorded as events in the survival analysis.

(B) FACS detection of ^PBMC15^MCAR-NKT and MCAR-T cells in various tissues of experimental mice terminated on day 60. Data are related to Figures 5E and 5F.

(C-F) Dose-gradient comparison among ^Allo15^MCAR-NKT, ^PBMC15^MCAR-NKT, and MCAR-T cells in an H226-FG human MPM xenograft mouse model. Therapeutic cells were administered via peritumoral injection. (C) Experimental design. (D) Tumor size measurements over time. Sample sizes (n) are indicated in the figure. (E) Tumor size measurements on day 35. (F) Tumor weight measurements on day 35.

(G) FACS detection of cytotoxic molecule (i.e., Perforin and Granzyme B) production by the indicated therapeutic cells. Data are related to Figures 5J and 5K.

Representative of 2 experiments. Data are presented as the mean ± SEM. ns, not significant; ****p < 0.0001 by log rank (Mantel-Cox) test adjusted for multiple comparisons (A).

**

**

**Fig. S6. Studying the *in vivo* antitumor efficacy of ^Allo15^MCAR-NKT cells in MSTO-FG and H226-FG metastatic human MPM xenograft NSG mouse models; related to Fig. 6.**

(A) Experimental design. In one experiment, mice were monitored for tumor progression until day 22, whereas in a separate experiment, mice were euthanized on day 15 to evaluate the immune checkpoint profiles of the therapeutic cells.

(B) BLI images measuring tumor loads in experimental mice over time.

(C) Quantification of (B) (n = 3).

(D) FACS analyses of immune checkpoint expression on the indicated therapeutic cells collected from the lungs of experimental mice on day 15 (n = 5).

(E) BLI images measuring tumor loads in experimental mice over time. Data correspond to Figure 6J. Mouse #2 from the Vehicle group, mouse #2 from the ^Allo15^MCAR-NKT cell group, and mouse #4 from the ^Allo15^MCAR-NKT+anti-TIM-3 group are shown in Figure 6J.

(F) Immunohistochemical (IHC) staining showing GFP⁺ H226-FG tumor cells and CD3⁺ T or NKT cells in the lungs of experimental mice. Tissue samples were collected on day 28. For each section, the same region was imaged to evaluate the distribution and infiltration of NKT or T cells into the tumor nodules.

Representative of 2 experiments. Data are presented as the mean ± SEM. ns, not significant; *p < 0.05; **p < 0.01; ***p < 0.001; ****p < 0.0001 by one-way ANOVA (D).

**

**

**Fig. S7. Studying the *in vivo* antitumor efficacy of ^Allo15^MCAR-NKT cells in an H226-FG metastatic human MPM xenograft NSG mouse model; related to Fig. 6.**

(A-C) *In vivo* kinetics of the three therapeutic cells. (A) Experimental design. (B) FACS detection of ^Allo15^MCAR-NKT, ^PBMC15^MCAR-NKT, and MCAR-T cells in peripheral blood of experimental mice over time. (C) Quantification of (B) (n = 5-6).

(D) FACS detection of ^Allo15^MCAR-NKT, ^PBMC15^MCAR-NKT, and MCAR-T cells in various tissues of experimental mice terminated on day 50. Data are related to Figure 6F.

(E) FACS analysis of chemokine receptor expression on therapeutic cells (n = 4-5). Expression of tissue inflammation–associated chemokine receptors was assessed by flow cytometry on ^Allo15^MCAR-NKT, ^PBMC15^MCAR-NKT, and MCAR-T cells isolated from the lungs of experimental mice euthanized on day 50.

(F) FACS detection of expression of immune checkpoints on the indicated therapeutic cells. Data are related to Figure 6G.

Representative of 2 experiments. Data are presented as the mean ± SEM. ns, not significant; *p < 0.05; **p < 0.01; ***p < 0.001; ****p < 0.0001 by one-way ANOVA (E).

**

**

**Fig. S8. ^Allo15^MCAR-NKT cells exhibit low GvHD risk and minimal CRS characteristics; related to Fig. 8.**

(A-D) Studying the GvHD risk of ^Allo15^MCAR-NKT cells using an NSG-MHC I/II DKO xenograft mouse model; related to Fig. 8, D to I.

(A) Experimental design. (B) Clinical GvHD score (n = 5). (C) Body weight change (n = 5). (D) Kaplan-Meier survival curves (n = 5).

(E and F) All three therapeutic cells demonstrated robust tumor-killing activity in an H226-FG human MPM xenograft intraperitoneal model; related to Fig. 8, J to M.

(E) BLI images measuring tumor loads in experimental mice over time. (F) Quantification of (E) (n = 3).

Representative of 3 experiments. Data are presented as the mean ± SEM. ns, not significant; ****p < 0.0001 by one-way ANOVA (F), or by log rank (Mantel-Cox) test adjusted for multiple comparisons (D).





**Fig. S9. ^Allo15^MCAR-NKT cells exhibit a high safety profile; related to Fig. 8.**

(A) ELISA quantification of mouse IL-6 and SAA3 secretion in mouse peritoneal fluid collected on Day 13 (n = 3); related to Fig. 8J-8M.

(B) ELISA analyses of human cytokine production in mouse serum collected on Day 13 (n = 3).

(C) ELISA quantification of organ damage markers in mouse serum collected on the indicated days.

(D-G) Studying the toxicity of ^Allo15^MCAR-NKT cells against mouse HSPCs using a human xenograft NSG mouse model. (D) Experimental design. On day 15, mouse bone marrow (BM) cells were collected and subjected to flow cytometry. (E) FACS detection of mouse long-term HSPCs (LT-HSPCs; gated as Sca1^+^c-Kit^+^ SLAM^+^ cells), short-term HSPCs (ST-HSPCs; gated as Sca1^+^c-Kit^+^Flt3^-^SLAM^-^ cells), and multi-potent progenitor cells (MPPs; gated as Sca1^+^c-Kit^+^ Flt3^+^SLAM^-^ cells) in BM cells collected from the NSG mice receiving ^Allo15^MCAR-NKT cell treatment. (F) Mouse HSPC killing data by ^Allo15^MCAR-NKT cells (n = 3; n indicates different mice). (G) FACS plots showing the lack of mouse CD1d expression on mouse HSPCs.

(H-L) Studying the toxicity of ^Allo15^MCAR-NKT cells against CD34^+^ HSPCs purified from G-CSF-mobilized healthy donor leukopak. (H) Diagram showing the CD34^+^ HSPC collection from G-CSF-mobilized healthy donor leukopak. (I) FACS detection of LT-HSPCs, ST-HSPCs, and MPPs in the G-CSF-mobilized healthy donor CD34^+^ HSPCs. (J) FACS plots showing the lack of CD1d expression on G-CSF-mobilized healthy donor CD34^+^ HSPCs. (K) Experimental design to study human HSPC killing by ^Allo15^MCAR-NKT cells. G-CSF-mobilized healthy donor CD34^+^ HSPCs were mixed with ^Allo15^MCAR-NKT cells at 1:1 ratio and cultured *in vitro* for 24 hours. (L) HSPC killing data at 24 h (n = 4).

Representative of 3 experiments. Data are presented as the mean ± SEM. ns, not significant; *p < 0.05; **p < 0.01; ***p < 0.001; ****p < 0.0001 by Student’s *t* test (F and L), or one-way ANOVA (A and B).





**Fig. S10. ^Allo15^MCAR-NKT cells can be engineered with an sr39TK suicide switch, enabling controlled depletion; related to Fig. 8.**

(A) Schematic showing the experimental design to generate allogeneic HSPC-engineered NKT cells equipped with a MCAR gene, and an sr39TK suicide gene (denoted as ^Allo^MCAR-NKT/TK cells). Lenti/iNKT-MCAR-sr39TK, lentivector encoding iNKT TCR, MCAR, and sr39TK.

(B) Studying the *in vitro* depletion of ^Allo^MCAR-NKT/TK cells via GCV treatment. GCV, ganciclovir that specifically depleting cells expressing the sr39TK gene. ^Allo^MCAR-NKT/TK cells were cultured *in vitro* in the presence of gradient concentrations of GCV for 3 days, followed by quantification of live cells via FACS analysis (n = 5).

(C-E). Studying the *in vivo* depletion of ^Allo^MCAR-NKT/TK cells via GCV administration. (C) Experimental design. (D) FACS detection of ^Allo^MCAR-NKT/TK cells from the indicated tissues on Day 5. (E) Quantification of (D) (n = 3).

Representative of 3 experiments. Data are presented as the mean ± SEM. ns, not significant; **p < 0.01; ****p < 0.0001 by Student’s *t* test (E), or one-way ANOVA (B).





**Fig. S11. Studying the immunogenicity of ^Allo15^MCAR-NKT cells.**

(A and B). Studying the T cell-mediated allorejection against ^Allo15^MCAR-NKT cells using an *in vitro* mixed lymphocyte reaction (MLR) assay. PBMCs from over 8 random mismatched healthy donors were used as responder cells, and irradiated ^Allo15^MCAR-NKT cells were used as stimulator cells. Data from three representative donors are presented. ^PBMC15^MCAR-NKT and MCAR-T cells were included as an allorejection control. (A) Experimental design. (B) ELISA analyses of IFN-γ production on day 4 (n = 4).

(C and D) Studying the NK cell-mediated allorejection against ^Allo15^MCAR-NKT cells using an *in vitro* MLR assay. PBMC-NK cells isolated from over 8 random mismatched healthy donors were used. Data from one representative donor are presented. ^PBMC15^MCAR-NKT and MCAR-T cells were included as allorejection controls. (C) Experimental design. (D) FACS quantification of the indicated live cells on day 0 and day 1 (n = 4).

(E and F). FACS measurements (F) and quantifications (G) of surface HLA-I/II and NK ligands (i.e., ULBP-1 and MICA/B) on ^Allo15^MCAR-NKT cells (n = 4; n indicates different cell product batches).

(G) scRNA-seq analysis of the indicated therapeutic cells. Violin plots showing the expression levels of HLA-related and NK ligand genes.

(H) FACS measurements of surface HLA-I/II and NK ligands (i.e., ULBP-1 and MICA/B) on the indicated therapeutic cells post *in vivo* antitumor response as described in Fig. 6A (n = 4; n indicates different experimental mice).

(I) Illustration depicting the hypoimmunogenecity working model of ^Allo15^MCAR-NKT cells.

Representative of 3 experiments. Data are presented as the mean ± SEM. ns, not significant; *p < 0.05; **p < 0.01; ***p < 0.001; ****p < 0.0001 by Student’s *t* test (D), or one-way ANOVA (B, F, and H).

**Table. S1. Gene list used to identify clusters in the scRNA-seq analysis.**

| **Cell clusters** | **Genes** |
| --- | --- |
| 1. Proliferating | *CENPF, NUSAP1, TPX2, UBE2C, CCNA2, CKS1B, CENPE, CDCA8, CDCA3, MKI67, SHCBP1, NEIL3, CDKN3, SPC24, 2810417H13RIK, CCNB2, HMMR, AURKB, FAM64A, BIRC5, RRM2, CENPM, CDCA5, SKA1, CDK1, KIF22, ESCO2, TACC3, TK1, PLK1, MXD3, STMN1, FBXO5, CEP55, CCNB1, ASF1B, HIST1H2AE, FIGNL1, CDC20, KIF23, E2F1, MCM5, RAD51, CDCA2, SPC25, HIST1H2AP, KIFC1, PRIM1, UHRF1, LMNB1, LIG1, NRM, TCF19, PRC1, DHFR, RRM1, NDC80, CENPW, DLGAP5, MCM7, KNSTRN, 1500009L16RIK, GMNN, LOCKD, MAD2L1, HMGB3, DIAPH3, CLSPN, HMGN2, HIST1H1B, CIT, PMF1, HMGB2, MCM3, H2AFZ, TYMS, SMC2, TOP2A, PTMA, INCENP, TIPIN, DUT, CENPA, TMPO, ORC6, CCDC34, CKS2, PPIA, CSRP1, HMGN5, CRIP1, RNASEH2B, FKBP2, NCAPD2, ANP32E, HELLS, MCM2, RFC5, LGALS1, DCTPP1, PLP2, FEN1, H2AFV, TUBA1B, NASP, RAN, BANF1, H2AFX, HAUS4, ACTG1, PIH1D1, PPIL1, MCM4, 2700029M09RIK, RACGAP1, EZH2, CDKN2C, LSM, PCNA, CLIC1, RBM3, DAP, S100A4, HMGB1, VIM, MCM6, DBI, RPA3, PFN1, ANP32B, NUCKS1, DEK, DNAJC9, S100A6, TUBB4B, DDX39, TPM4, CALM1, ITGB7, CMC2, TAGLN2, YWHAH, ACTB, S100A10, ANXA2, RANBP1, LRRC58, TXN1, CDC25B, 2700094K13RIK, UBE2S, COMMD1, GAPDH, CBFB, TUBB5, CFL1, SDF2L1, ANAPC5, SLBP, LBR, LGALS3, PRDX1, ATPIF1, HNRNPA3, RBBP7, HNRNPAB, NUDT21, SH3BGRL3, PYCARD, RFC2, RPS27L, CORO1A, CALM3, TUBA1C, SMC4, ACTR3, RAD21, EXOSC8, HNRNPA2B1, 1700097N02RIK, CAPG, PSMD13, REEP5, YWHAQ, MRPL18, S100A11, GLTP, SRSF2, PRELID1, BUB3, SMC6, CMTM7, HNRNPF, SNRPD1, DPY30, HPRT, ERH, TPM3, CD48, EMP3, NAP1L1, RHOA, COX5A, PSMA4, SUB1, SNRPE, RALY, SDHB, ATP5F1, H3F3A, PSMB9, HINT1, SRSF7, SRSF3, HSP90B1, GLIPR2, PPP1CA, CBX3, ANXA6, HDGF, PSMB2, ARPC5, MYL12A, COTL1, SERINC3, CAPZB, WDR1, COX7A2, HN1, YWHAE, COX5B, RBBP4, NDUFA4, IFI27L2A, XIST, ARL6IP1* |
| 2. Effector | *GZMB, PFN1, CD48, VIM, ZEB2, S100A4, S100A10, EMP3, RAP1B, KLF2, LGALS3, CX3CR1, CCL5, CRIP1, LGALS1, S100A6, CTSD, KLRC1, KLRD1, PYCARD, SELPLG, NA, S1PR5, LSP1, S100A13, S1PR4, RASGRP2, AHNAK, ATP1B3, CALM1, REEP5, NA, IL18R1, SPN, TXNDC5, GNA15, ANXA2, ZYX, ITGB7, PRR13, OSTF1, KLRK1, NA, TAGLN2, BORCS7, MYO1G, ANXA6, IFNGR1, GLIPR2, IER2, RASA3, NA, IL18RAP, KLF3, RACGAP1, FLNA, PIM1, NA, SGK1, SEPTIN11, TSC22D3, ATP2B1* |
| 3. Memory-like | *LEF1, TCF7, KLF2, TXK, BACH2, LTB, FLT3LG, TNFSF8, CMTM8, IL23A, TIMP1, WNT7A, CCR7, IL7R, IL6R, IFNGR2, SELL, MAL, EEF1A1, ACTN1, TRABD2A, TPT1, EEF1B2, NELL2, NOSIP, PABPC1, ZFP36L2, TSC22D3, CXCR4, ZFP36, BTG1, ANXA1, LMNA, CD55, TNFAIP3, FTH1, RGCC, GPR183, ZNF683, CCL5, IL32, GZMA, TNF, CKLF, ACTB, CD52, TRAF3IP3, SH3BGRL3, SIT1, S100A4, CISH, MYL12A, PTPRCAP, PFN1, CCR5* |
| 4. Exhausted | *RGS1, CD3G, CXCR6, CCL4, AW112010, CCL3, NR4A2, 2900026A02RIK, LAG3, ACTN2, SH2D2A, ABI3, PTGER4, MBNL1, ID2, ARL6IP1, CD244, OSGIN1, ADGRG1, 4-Sep, GZMB, LAX1, PTPN22, GZMA, PDCD1, PLAC8, GLRX, ISG15, VMP1, ABCB9, RGS3, FGL2, FASL, EFHD2, SERPINA3G, IFI47, IFIT1, RBPJ, ETV1, TOX, ZBED2, TOX2, CXCL13, TNFSF4, FAM3C, GZMB, CSF1, CCL3, CD70, IFNG, NAMPT, FASLG, IL2RA, CXCR6, CD74, IL2RB, IL2RG, TNFRSF9, LAYN, ENTPD1, HAVCR2, CTLA4, KRT86, TNFRSF18, GEM, TIGIT, DUSP4, TOX, HAVXCR2* |
